# Expertise-dependent visuocognitive performance of chess players in mating tasks: evidence from eye movements during task processing

**DOI:** 10.3389/fpsyg.2024.1294424

**Published:** 2024-10-24

**Authors:** Thomas Küchelmann, Konstantinos Velentzas, Kai Essig, Thomas Schack

**Affiliations:** ^1^Department of Neurocognition and Action-Biomechanics, Faculty of Sport Sciences, Bielefeld University, Bielefeld, Germany; ^2^Center of Cognitive Interaction Technology, Bielefeld University, Bielefeld, Germany; ^3^Faculty of Communication and Environment, Rhine-Waal University of Applied Sciences, Kamp-Lintfort, Germany

**Keywords:** visual attention, eye tracking, perceptual processing, multi-sensor observation, chess expertise

## Abstract

**Introduction:**

Visuocognitive performance is closely related to expertise in chess and has been scrutinized by several investigations in the last decades. The results indicate that experts’ decision-making benefits from the chunking process, perception and visual strategies. Despite numerous studies which link these concepts, most of these investigations have employed common research designs that do not use real chess play, but create artificial laboratory conditions via screen-based chess stimuli and obtrusive stationary eye tracking with or without capturing of decision-making or virtual reality settings.

**Methods:**

The present study assessed the visuocognitive performance of chess novices, intermediates and experts in a real chess setting. Instead of check detection, find-the-best-move tasks or to distinguish between regions of a chessboard that were relevant or irrelevant to the best move in previous studies, we introduced n-mate tasks and sequentially manipulated their difficulty. Due to the complexity of the tasks, we monitored players’ visual strategies in a fine-graded initial phase (different time intervals instead of analysing a fixed number of first fixations) of task-solving and for complete trials, employing non-obtrusive mobile eye tracking, multi-sensor observation and full-automatic annotation of decision-making.

**Results:**

The results revealed significant expertise-dependent differences in visuocognitive performance based on a circumstantial spatial and temporal analysis. In order to provide more detailed results, for the first time the analyses were performed under the special consideration of different time intervals and spatial scalings. In summary, experts showed a significantly higher number of fixations on areas of interest and empty squares between pieces in the task processing than less-skilled players. However, they had a strikingly low total number of fixations on the whole board and in complete trials.

**Discussion:**

As a conclusion, experts apply different visual search strategies in problem-solving. Moreover, experts’ visuocognitive processing benefits from stored chunks of mating constellations.

## Introduction

The term “expertise” has become part of everyday language and is mostly associated with a high level of education and social reputation. A more precise domain-specific definition is that an “expert” should give proof of reproducible performance on representative domain-specific tasks using standardised tests (Ericsson and Smith, 1991). Considering sport and exercise as an example, *expertise* is defined as the ability to consistently demonstrate superior athletic performance (Janelle and Hillman, 2003; Mann et al., 2007). Perceptual and cognitive skills play a crucial role here. Schack et al. (2014) link practitioners’ performance to perceptual and cognitive skills to help athletes identify and acquire environmental visual information and integrate them with existing knowledge to select and execute appropriate actions (Marteniuk, 1976).

Perceptual and cognitive abilities are prerequisites for high-level expertise also in tasks which focus on pattern recognition (Chase and Simon, 1973a,Chase and Simon, 1973b; Gobet and Simon, 1996; Charness et al., 2001). According to Campitelli (2017), cognitive processes in chess imply numerous cognitive subfunctions and abilities as “pattern recognition, attention, memory, imagery, thinking, and decision-making.” The author points out that the majority of those processes is highly correlated with the visual perception and the visual data collection strategies. They are subdivided into selecting/responding to relevant stimuli and the recognizing of key information. Hence, perceptual and cognitive skills are conceptually summarized by the term of *visuocognition* (Warren, 1993).

Additionally, Campitelli refers to the PPP model (Campitelli et al., 2014) which implies plasticity, chess chunks/templates-based pattern recognition, and chess heuristics. This model assumes that chess skill is a trait developed by domain-specific pattern recognition and heuristic processes. Given that the term visuocognition implies a variety of different perceptual and cognitive functions, it is reasonable to assume that chess performance depends on them (e.g., Kiesel et al., 2009; Sheridan and Reingold, 2017). However, the meta-analysis by Burgoyne et al. (2016) has shown that the existing findings are firstly inconsistent and secondly that more specific research is needed in order to clarify the complex concept of visuocognition.

In order to understand how visuocognitive proficiencies are developed to reach high performance, numerous studies indicate that intense practice is required (Galton, 1979; Ericsson, 1996; Ericsson and Lehmann, 1996). Moreover, dedicated practice can raise visuocognitive skills to an expert level and peak performance (Ackerman and Cianciolo, 2000).

From this point of view, chess has become a distinguished object of research concerning pattern recognition, visuocognition and decision-making. It is a popular and highly competitive expert domain, referred to as the *“Drosophila of Artificial Intelligence”*—a prototype for many research in expertise field (Simon and Chase, 1973; McCarthy, 1990). This may be due to the distinct rating system based on tournament performance (Elo, 1978) and the extremely high state-space complexity. In fact, from the starting position about 10^47^ legal game positions can be reached (Shannon, 1950), despite of its easily controllable setting (e.g., material, space and time control). Consequently, the enormous complexity of strategic elements and decision-making processes (Gobet and Charness, 2006) forces chess players to acquire a tremendous amount of knowledge far beyond the basic rules of the game.

## Background and related work

Numerous different approaches in chess research address the question of how humans achieve, maintain and improve high levels of chess expertise, indicating that visuocognitive processing and chunking are crucial aspects (De Groot, 1966, De Groot, 1978; Chase and Simon, 1973a,Chase and Simon, 1973b; Reingold and Sheridan, 2023).^1^

One of the earliest concepts, considered to understand chess players’ visuocognitive processing, is the *working memory*—a central concept in human cognitive functioning (Logie et al., 2020). The framework of De Groot (1966) is regarded as the origin of research about chess memory skills and a milestone of chess research. He initially assumed that grand masters appear to have a superior working memory capacity (e.g., planning long move sequences), innate talent and surpassing general intelligence. To test this hypothesis, he measured the working memory capacity of chess experts employing recall tasks and think aloud protocols during decision-making (De Groot, 1978) in problem-solving by six grand masters (the best experts), four candidate masters (experts), two women’s champions of the Netherlands (experts), five hoofdklasse players (experts in the USCF rating system) and five weak hoofdklasse players to second class players (class A to C players in the USCF rating system). Surprisingly, the analysis of the think aloud protocols revealed a similar number of moves for both grand masters, experts and weaker experts. This outcome indicates that there are only slight depth-of-search effects as a function of skill. However, grand masters were faster to identify more efficient moves than candidate masters. In the recall task, grand masters performed significantly better than candidate masters and weaker experts when memorizing meaningful chess constellations. Despite criticism regarding the participants’ selection and the small sample size, De Groot’s work can be seen as a “*beacon*” for chess and memory research.

As a consequence of De Groot’s findings, Chase and Simon (1973b) aimed to identify what kind of memorized domain-specific patterns experts may benefit from and employed memorization tasks with meaningful versus random chess positions presented to a master, a class A player and a beginner. As a result, the expert only outperformed the less skilled players at memorizing meaningful chess constellations. They link these findings to the *chunking theory* (Simon and Gilmartin, 1973).

In summary, the results of De Groot (1966, 1978) and Chase and Simon (1973a,b) provided a clearer understanding of the essence of visuocognition as they indicated that grand masters simply circumvent the limitations of their working memory capacity (i.e., through practice and learning domain-specific knowledge).

To gain deeper insights into the performance and visuocognitive advantages of chess experts Reingold et al. (2001b) assessed the inhibition of irrelevant information for 14 novices, 14 intermediates and 14 experts. They employed a screen-based reaction time experiment with a king being checked by either a cued or a non-cued attacker. Here, experts had problems inhibiting irrelevant information (not cued pieces) and revealed an interference effect. Novices, on the other hand, did not. In the case of cued checking pieces experts showed a faster reaction time than novices. This result indicates that in checking constellations, experts also benefit from task-specific automaticity, besides visuocognitive advantages (*η^2^* = 0.17). Consequently, the processing of subliminal visual stimuli is an important aspect of visuocognition.

From this point of view, Kiesel et al. (2009) investigated the visuocognitive processing of 12 chess players and 24 chess novices by assessing subliminal response *priming* effects. They employed subliminal stimuli of static check versus no check and nonsensical stimuli. Each target was preceded by a prime which was congruent or incongruent with the target. Hence, congruent primes pre-activate the requested response to the target but incongruent primes do the opposite (Dehaene et al., 1998; Eimer and Schlaghecken, 2002). The results indicate that experts benefit from congruent priming only in the case of check versus no check stimuli (reported main effect: *η^2^* = 0.62), whereas novices, do not. The authors attribute the experts’ priming effects to chunks of checking constellations as the nonsensical stimuli do not correspond to any chunks.

Similarly, Postal (2012) and Küchelmann et al. (2022) explored subliminal response priming effects in more detail by employing screen-based reaction time settings. Postal (2012) assessed 8 experts, 9 intermediates and 9 novices. They showed that only intermediates benefited from *attentional* priming combined with cued attackers (reported main effect: *η^2^* = 0.24). The results are in line with those of Reingold et al. (2001b) and Kiesel et al. (2009). Küchelmann et al. (2022) extended the design of the Kiesel et al. (2009) by assessing not only novices and experts but also intermediates. Moreover, target and task complexity were gradually increased and priming duration was varied. The results of Küchelmann et al. (2022) support previous findings on the perceptual superiority of experts through increased stimulus and task complexity (reported effects: *η^2^* = 0.22., *η^2^* = 0.21, *η^2^* = 0.17). The authors argued that due to stored chunks of checking and mating constellations the detection and anticipation of potential threats to the king is rooted in experts’ more efficient visuocognition.

The underlying processes of visuocognition may mirror the inner structure of a black box from the behaviourism perspective (Friedenberg and Silverman, 2006). Visual information processing is a crucial aspect of visuocognition and can be investigated in detail by *eye tracking*—a sensor technology which detects what a person is looking at in real-time by collecting information such as central view, gaze vectors and pupil position. As a diagnostic procedure, eye tracking allows the analysis of visual pathways during perceiving information (Holmqvist et al., 2011; Mele and Federici, 2012).

Different eye-tracking devices are developed, according to the methodology and the research objective, aiming to achieve the highest reliability and usability. For example, screen-based remote eye tracking bars are applied to analyse visual scan paths during the presentation of specific information on a screen (i.e., a specific chess constellation). On the other hand, eye tracking glasses are developed to support the spatial mobility of participants during data collection. Such mobile eye trackers are used in real action situations in sports as well as in laboratory environments, providing higher usability and comfort. Furthermore, mobile eye tracking offers a high reliability, despite of participants’ head and body movements (Mussgnug et al., 2015; Narcizo et al., 2013).

Reingold et al. (2001a) applied eye tracking to measure skill-related differences in visual search behaviour of chess experts and intermediate players. The given task for 16 novices, 8 intermediates and 8 experts was to decide for the best move in static chess positions (for a review please see Reingold and Charness, 2005). Experts revealed fewer fixations per trial and greater amplitude saccades than intermediates (reported main effects: *η^2^* = 0.40, *η^2^* = 0.30). However, no differences in fixation duration were observed. Moreover, experts placed a larger percentage of fixations *between* pieces rather than on the pieces themselves. Also, Charness et al. (2001) examined the spatial distribution of the first five fixations of 12 intermediates and 12 experts when choosing the best move in five chess positions. The findings are in line with the results in Reingold et al. (2001a), indicating that, in comparison to intermediates, experts demonstrated a greater tendency to fixate on empty squares in each position. This expertise-dependent saccadic selectivity supports Chase and Simon’s (1973a,b) chunking hypothesis as experts’ visual search strategies focus on chunks rather than on pieces.

Furthermore, this conclusion is strengthened in the Sheridan and Reingold (2014) eye tracking study which involved 17 experts and 24 novices and showed that experts outperformed novices in rapidly discriminating relevant and irrelevant regions in find-the-best-move tasks (reported main effects: *η^2^* = 0.23, *η^2^* = 0.29, *η^2^* = 0.67). In the same line of reasoning, Sheridan and Reingold (2017) investigated 16 experts’ and 23 novices’ ability to distinguish possible and impossible sequences of moves (one to three moves of the knight piece). The eye tracking analysis revealed that experts used more efficient visual search strategies for identifying relevant move sequences (reported main effects: η^2^ = 0.13, η^2^ = 0.22, η^2^ = 0.11, η^2^ = 0.30).

Nevertheless, it is still unclear whether visual-perceptive processes (symbols of pieces) as a component of visuocognition in chess or logical deductive processes (letters representing pieces) are crucial. In latest research, Reingold and Sheridan (2023) represented pieces both by symbols and letters in stimuli which 18 experts and 24 novices had to scan for a double checking. The results indicate that experts benefit primarily from immediate visual-perceptive processes (reported main effect: η^2^ = 0.16).

Strikingly, most studies utilize check-versus-no-check and find-the-best-move tasks and focus on screen-based designs or artificial virtual reality (VR) settings (Hainke and Pfeiffer, 2021) which only imitate close-to-natural chess environments (e.g., chess play in tournaments). For instance, Hainke and Pfeiffer (2021) assessed 14 experts and 19 novices in a VR chess environment and took the cut off interval of the first 5 s for the analysis of the gaze behaviour into account. As a result, experts gazed significantly (*p* < 0.01) more at free positions (on average 55% of the task time and only 45% of the time at pieces), while novices spent significant (*p* < 0.01) more time gazing at pieces (53% of the time). In terms of visuocognitive processing, there are promising approaches that combine Augmented Reality with capacitive 3D printed objects (Günther et al., 2018; Kim et al., 2017). They demonstrate that chess players can participate in remote interaction while having the same tactile experience as in co-located scenarios. Moreover, immersive VR programs combined with head-mounted displays and eye tracking are a powerful tool (e.g., for the detection, vision screening and rehabilitation of vision problems [Dæhlen et al., 2023]). However, the lack of depth perception in VR has been criticized (e.g., Prakash et al., 2016) and head-mounted interfaces may result in greater distance from natural chess play. The present study addresses the question of how to assess visuocognitive performance under close-to-tournament conditions and with annotation of decision-making and points of interest. Contrary to remote eye tracking or eye tracking with a chin rest which is preferably applied to screen-based settings (Niehorster et al., 2017), we employ mobile eye tracking. For a more differentiated analysis, we address among others, the question to which extent experts demonstrate visuocognitive superiority in the early stages (i.e., the first 2 to 5 s) of task processing.

## Contributions of this work

As an elaboration of De Groot’s (1966, 1978) studies, we used the apparatus of Küchelmann et al. (2017). Following De Groot’s research goals and extending Küchelmann’s design (Küchelmann et al., 2022), the main aim of the present study was to analyse visual search strategies as part of visuocognitive functions according to different chess expertise (novice, intermediate, expert) and task difficulty (n-mate tasks). Supplementary to Küchelmann et al. (2022), a strict grading of task difficulty and complexity was applied in order to perform a more detailed analysis (n-mate task combinations and restricted number of moves to the solution). Herein, we measured incremental changes of players’ visuocognitive performance during problem-solving in detail, taking into account pre-defined sequences of Areas Of Interest (*AOI*s), expertise and decision-making timeline (i.e., the first seconds of problem-solving and whole trials). Aiming to scrutinize expertise as a decisive performance factor of chess, we manipulated the task difficulty. In a close-to-natural chess environment, players’ eye movements were recorded using mobile eye tracking glasses and their decision-making was fully automatically annotated (Küchelmann et al., 2017). The study design circumvented the application of abstract two-dimensional chess diagrams and allowed players more freedom of body movement and view angles. Therefore, it puts into practice more distance from artificial laboratory conditions. Eye tracking data and visual search strategies can be determined as part of the personality and performance of chess players. As a consequence, our eye tracking data and data from further implementations (future work) can be used to optimize virtual players in terms of Artificial Intelligence. Consequently, in the future, an extension of the current data collection (i.e., more participants and/ or cognitive pre-activation, psyching-up and psyching-down) could be used in the future to facilitate assistive systems to support individualised training—visual guidance procedures—and augmented feedback, while improving the algorithms of virtual players (Dhou, 2018). All this could lead to a cost-effective cognitive processing and improved performance (decision-making, self-efficacy and self-confidence, Kővári and Katona, 2023). Moreover, the data from this study can facilitate the construction of convolutional neural networks which predict not only the visual attention but also the response of chess players (Le Louedec et al., 2019). Furthermore, the multimodal analysis of visuocognition can be applied to several scientific fields such as education and information technologies, learning, reading and writing. In combination with additional sensors such as brain-computer interfaces (Saha et al., 2021) and EEG, this may also apply to assistive systems for people with motor disabilities (Gannouni et al., 2022) and to industrial work processes.

## Materials and methods

We employ the apparatus described in Küchelmann et al. (2017) which puts the full-automatic analysis of chess moves and eye tracking data in practice without the need for an error-prone and time-consuming manual data annotation.

### Study design

#### Dependent variables

The present study was based on a 3 × 3 design (expertise × task difficulty). The dependent variables are the correctness of answers, the task processing time (i.e., board presentation until the replacement start of a piece), the absolute number of fixations during the task processing time, fixations’ duration and the relative number of fixations on empty squares and on the pre-defined AOIs. According to the fact that the processing time varies between participants and groups, we undertook a data normalization for the absolute number of fixations on empty squares and on AOIs for complete trials. For this purpose, we divide the absolute number of fixations for each participant by the processing time (in seconds) and these fractions were multiplied by 100.

#### Tasks

**Figure 1:**
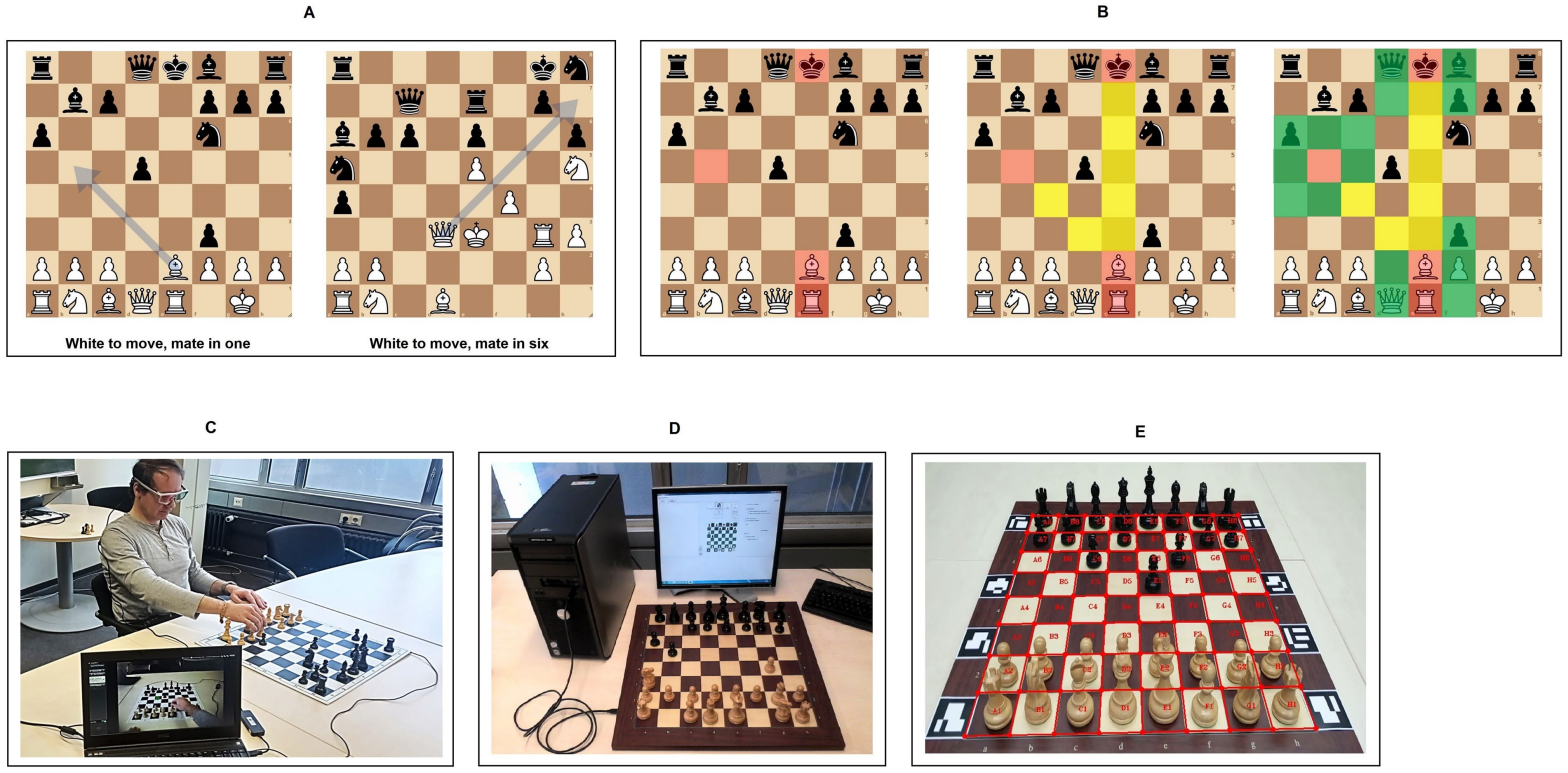
**(A)** Examples for n-mate tasks: Task 1 “White to move, mate in one” and Task 11 “White to move, mate in six”; **(B)** Three different types of AOIs—explained on the basis of Task 1. Left side: AOI 1 (red) includes the attacked king, both attackers bishop on e2 and rook on e1 and the target square b5 of the bishop’s attacking move; middle: AOI 2 includes AOI 1 plus the bishop’s piece replacement squares (yellow) and squares X-rayed (yellow) by the rook; right side: AOI 3 includes AOI 1 and AOI 2 plus the nearest neighbour squares (green) of all AOI 1 squares; **(C)** Experimental setting demonstration: Chess player whose gaze is recorded by SMI eye tracking while solving Task 1; **(D)** The DGT e-board connected to a screen and a computer via USB; **(E)** Implementation of eight ArUco markers to stabilise the chessboard detection.

The task is to detect a mate in a number of moves under time pressure (maximum processing time: 150 s). A total of eleven n-mate detection tasks were selected based on online chess puzzles from the open-source internet chess server Lichess (2010) (lichess.org). The task difficulty increases sequentially, starting with a mate in one and ending with a mate in six moves (see Figure 1A), and always presented in the same order. The sequential increase in difficulty was guaranteed by the ranking of the training tasks on Lichess (2010) (lichess.org) and ensured by the assessment of two experts (ELO rating 2050 and 2100) who independently ranked the first four tasks as “easy,” the following four tasks as “medium” and the final three tasks as “difficult” with a rank correlation of 0.91. The selection of 4 × 4 × 3 (trials) for the degree of difficulty conditions (easy × medium × difficult) was made following the assumptions that: Firstly, the difficulty is combined with a cognitive overload for novices and intermediates. Secondly, an expand of the trials for the difficult condition could cause noise due to the lack of concentration and finally, under consideration of the fact that the difficult condition requires intensive strategical planning and required more moves for each trial, we attend to balance the needed time between all conditions.

#### Phases of task processing

Aiming to provide a more detailed analysis, we pre-defined three different *phases of task processing*. The first phase, referred as *reasoning part*, was determined through the task start signal (activating the time registration and the eye tracking recording) and the elevation of a particular chess piece from the electronic chessboard by the participant which automatically registered the split time of the current trial and marked the end of eye tracking record (Küchelmann et al., 2017).

The end of the reasoning part automatically defined the beginning of the *handling part*. In this phase, each participants’ action (e.g., piece replacement) was automatically registered, combined with a needed split time, by the electronic chessboard. All these data were accessible as a .txt file giving us at the same time the opportunity to control the response correctness. Based on the fact that contradictory and inconsistent approaches were taken in previous studies [e.g., analysis of the first five fixations in Charness et al., 2001 and analysis of the time window of the first 5 s in Hainke and Pfeiffer, 2021], we decided to specify the initial phase by the cut into a sequence of time windows (i.e., the first 2, 3, 4 and 5 s of each complete trial). The corresponding cut-offs were identified by the time stamp of the eye tracker.

#### Areas of interest

Arguing that chunks imply visual processing, not only pieces and empty squares were defined as AOIs but also three other hierarchically structured regions of the board were specified as AOI 1, AOI 2 and AOI 3 (see Figure 1B). AOI 1 consists of the attacked king, the attacking piece(s) and the target square(s) of the attacker(s). Therefore, it focuses only on the immediately relevant aspects of the attack on the king. AOI 2 consists of AOI 1 plus the piece replacement squares (mainly empty squares) representing the trajectories of the attacking move(s). Hence, these trajectories are therefore of interest in terms of planning the attacking move(s). AOI 2 consequently represents a more dynamic perspective which reflects the task. Finally, AOI 3 is an extended area which is constructed by extending AOI 2 by all the nearest neighbour squares of those squares which constitute AOI 1. We took into account that experts who processed meaningful (not random) chess positions in Reingold et al. (2001a) demonstrated a larger visual span than lower-skilled players and estimated that all squares and pieces outside of AOI 3 are outside of experts’ visual span. We therefore assume that the exterior of AOI 3 is completely irrelevant for the respective task. This in turn means that fixations on AOI 3 indicate at least a rudimentary understanding of the task, whereas fixations outside of AOI 3 indicate a lack of understanding or focus.

#### Hardware

In order to execute the recording of eye movements the non-invasive, wireless mobile and binocular Eye Tracking Glasses (ETG-2) from Sensomotoric Instruments^®^ (1991) (SMI) was employed (Figure 1C).The eye tracker calculates where the gaze is located in space relative to a frontal scene camera with a resolution of 960×720 pixels with 30 fps. The device calculates the gaze direction, based on coordinates computed through the analysis of the centre of the pupil and corneal reflection points. For this purpose, an infrared-cam with a light source is integrated into the eye tracking device and directed to participants’ eyeball. Simultaneously, an algorithm estimates the distance between the two reference points and transfers the gaze direction on the perceived scenes, captured by the high-definition frontal cam (which in our case recorded the chessboard). The sampling rate of the glasses is up to 120 Hz which provides a gaze tracking accuracy of 0.5° over all distances. The eye tracking data were processed on a DELL^®^ Precision 4,800 Laptop (15inch HD Screen).

An electronic chessboard (*e-board*) from DGT (Digital Game Technology^®^, 1993, see Figures 1D,E) was used to transmit real-time game data via USB to a DELL^®^ Latitude E6520 notebook (core 8 with an external HD screen 17inch with 100 Hz). The board design is standard tournament design: 55 × 55 mm tournament size squares, 8 mm board thickness and classical wood piece design and size. The e-board implements the mapping of each piece’s identity to its actual position on the chessboard and carries out the transcription of all moves. It was combined with ArUco marker detection (Garrido-Jurado et al., 2014; Bradski and Kaehler, 2008; OpenCV 3.1.0, detection of ArUco markers, 2015) in order to stabilize the board detection (see Figure 1E) with respect to perspective overlaps and occlusions (e.g., by participants’ arms and hands [see Küchelmann et al., 2017 for details]).

#### Software

The eye tracking data was processed using the BeGaze^®^ (2010) and IView^®^ software.

#### Participants

A total of 58 male chess players (*M*_age_ *=* 29.40, *SD*_age_ *=* 11.85) participated in the present study. With the assistance of G*Power, the required effect size for our criteria was determined to be *F* = 0.53, which ensures the high reliability (power = 0.95) of the findings presented in this study. A main criterion for the participation was that participants had normal vision (i.e., no contact lenses or glasses), because both can interfere with the eye tracker’s functionality by reflecting infrared light. None of them had any experience playing blindfolded chess, an important feature as we were investigating gaze behaviour.

For the underlying expertise classification players’ ELO or DWZ^2^ ratings were taken into account. An ELO/ DWZ score of 1.850 and above is considered an “expert” (class A player and better) and between 1.200 and 1.850 an “intermediate” (class D to class B player), respectively. Participants with an ELO or DWZ below 1.200 were classified as “novices” (class E and below class E player). In addition, players were classified as “novices” if they had no ELO or DWZ rating and reported having played a minimum of 20 and a maximum of 100 chess matches in their lifetime.

The first group consisted of “experts” (*N*_exp_ *=* 17, *M*_age_ *=* 33.53, *SD*_age_ *=* 13.93), the second group of “intermediates” (*N*_int_ *=* 20, *M*_age_ *=* 28.00, *SD*_age_ *=* 11.38) and finally the third group of “novices” (*N*_nov_ *=* 21, *M*_age_ *=* 27.38, *SD*_age_ *=* 9.17). All participants worked on the eleven mate detection tasks consecutively in the same order and looked at the board from white’s perspective (first row of the board). They were given informed consent before data collection.

#### Stimuli and procedure

Prior to data collection, all participants adjusted the chair height and distance from the board (50 cm) to ensure a clear view of the board (Figure 1C). Rapid movements of the head and upper body had to be avoided. Therefore, the instruction was to sit upright.

The next step was to use a three-point calibration for the eye tracking glasses. Each of the mate detection tasks was then set individually by the instructor while the participant was out of sight of the board to prevent him/ her from thinking about the task in advance. Then a start signal, which also started the time registration, told the participant to look at the board and start thinking.

Participants first had to look at the task in order to find a solution (*reasoning part*). Once the participant was sure to have found a solution, he or she had to move the piece(s) accordingly. Herein, the beginning of the (first) move simultaneously terminated the reasoning part and initiated the *handling part* (Figure 1C).

At the end of the data collection, a manipulation check (post-experimental questionnaire) was performed in order to see if participants felt distracted by the use of the eye tracking glasses.

## Results

Prior to the main analysis the manipulation check has shown that none of the participants felt distracted by the eye tracking, the duration of the experiment and the complexity of the setting. We performed a multivariate analysis (MANOVA) and the results show a significant effect with *F*(9,18) = 8.508, *Wilk’s λ* = 0.144 and *p* < 0.001, so separate univariate ANOVAs were calculated for each dependent variable (Tabachnick and Fidell, 1989).

For all performed analyses alpha level was set at 5% (*α* = 0.05). For all significant results Tukey post-hoc tests were performed and the eta squared (*η^2^*) was calculated as well as the achieved power, based on SD’s *σ*.

### Variations in correctness, processing time, numbers and duration of fixation

#### Correctness of solutions

It was hypothesized that for all task blocks (easy, medium and difficult tasks) experts would perform better than novices and intermediates in terms of number of correct solutions (*CS*), while intermediates would perform better than novices.

We took CS for each task block and for each expertise group as evidence of performance (see Table 1) and employed a Chi-Square Test (df = 10) with CS as the dependent variable. The Chi-Square test indicates that for all blocks of tasks (Table 1) intermediates have a better performance than novices and experts have a better performance than novices and intermediates (*Pearson Chi-Square* = 348.00, *p <* 0.001). This supports our hypothesis.

**Table 1:** Overview of the descriptive statistics of DF (duration of fixation), NF (number of fixations), PT (processing time), CS (correctness of solution), RNFES (relative number of fixations on empty squares), RNFAOI 1 (relative number of fixations on AOI 1), RNFAOI 2 (relative number of fixations on AOI 2) and RNFAOI 3 (relative number of fixations on AOI 3) for all task blocks and all expertise groups, taking into account complete trials.

Group	Task block	Mean DF [ms]	SD	SE	Mean NF	SD	SE	Mean PT [ms]	SD	SE	CS [%]	Mean RNFES	SD	SE	Mean RNFAOI 1	SD	SE	Mean RNFAOI 2	SD	SE	Mean RNFAOI 3	SD	SE
Experts	Easy	214.89	55.45	6.72	36.46	27.69	3.36	12,991.37	11,084.89	1344.24	97.05	193.57	77.36	9.38	47.92	37.04	4.49	107.69	59.36	7.20	234.01	80.84	9.80
Intermediates		222.11	32.76	3.66	132.11	124.69	13.94	41,204.65	34,852.04	3896.58	86.25	169.57	58.97	6.59	57.51	32.61	3.65	118.86	44.40	4.96	259.04	68.89	7.70
Novices		220.94	40.78	4.45	256.89	147.25	16.07	80,733.62	42,496.47	4636.74	57.14	163.63	49.54	5.41	51.74	24.35	2.66	113.48	53.44	5.83	251.04	60.98	6.65
Eperts	Medium	215.98	62.01	7.52	93.15	88.58	10.74	28,739.20	24,408.81	2960.00	98.53	222.17	74.98	9.09	77.43	62.74	7.61	154.23	90.49	10.97	269.79	73.76	8.94
Intermediates		240.34	45.91	5.13	236.50	159.41	17.82	77,162.01	45,879.37	5129.47	56.25	177.20	81.15	9.07	76.22	58.19	6.51	122.89	59.37	6.64	261.28	85.18	9.52
Novices		244.13	42.22	4.61	364.52	122.61	13.38	116,528.91	30,436.79	3320.93	28.57	177.12	77.88	8.50	68.17	30.40	3.32	119.70	43.18	4.71	239.67	59.39	6.48
Experts	Difficult	217.24	63.48	8.89	309.55	138.18	19.35	99,199.51	41,404.46	5797.78	60.78	156.32	59.41	8.32	70.21	43.45	6.09	213.32	57.28	8.02	300.05	70.15	9.82
Intermediates		222.32	33.63	4.34	417.37	101.31	13.08	130,624.75	18,216.20	2351.70	25.00	160.71	41.77	5.39	72.04	35.91	4.64	199.84	31.56	4.07	282.70	60.02	7.75
Novices		229.00	38.38	4.84	445.56	67.00	8.44	134,127.77	15,686.07	1976.26	3.17	166.28	31.04	3.91	65.38	29.08	3.66	175.19	41.67	5.25	277.84	46.98	5.92

#### Processing time

It was anticipated that experts’ and intermediates’ processing time (*PT*) would be significantly lower than the PT of novices for easy and medium tasks as experts and intermediates should be able to identify relevant pieces/squares faster (Chase and Simon, 1973a,Chase and Simon, 1973b). Regarding medium tasks, we also awaited the PT of experts to be significantly lower than the PT of intermediates. For the PT of difficult tasks, we expected significant differences between experts and intermediates as well as between experts and novices but not between intermediates and novices who both would be overextended by the difficult tasks.

**Figure 2:**
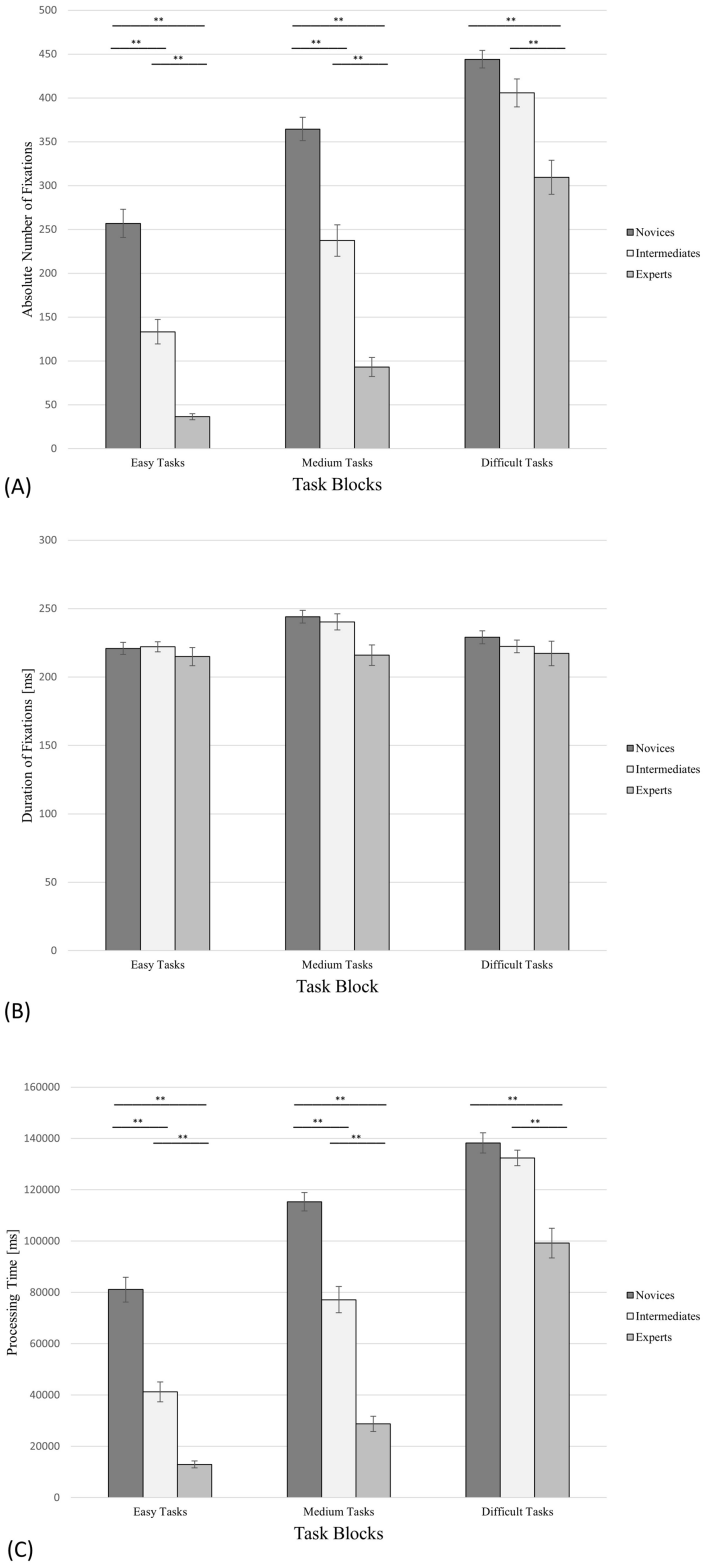
N-mate tasks, including all trials (correct solutions as well as incorrect or no answers) and the standard errors of the observed variables. **(A)** Number of fixations; **(B)** Duration of fixations; **(C)** Processing time. ** denotes “highly significant” (i.e., *p* < 0.01). *** denotes “extremely high significant” (i.e., *p* < 0.001).

We performed a one-way ANOVA (condition, expertise) on PT. The ANOVA showed a significant effect for PT in the case of easy tasks with *F*(2,55) *=* 49.65, *p <* 0.001 and *η^2^ =* 0.64. As hypothesised, the post-hoc analysis (Tukey LSD test) revealed significant differences between *PT*_exp_: *PT*_nov_ (*M*_exp_ *=* 12,991: *M*_nov_ *=* 80,733) and *PT*_int_: *PT*_nov_ (*M*_int_ *=* 41,204: *M*_nov_ *=* 80,733), but unexpectedly also between *PT*_exp_: *PT*_int_ (*M*_exp_ *=* 12,991: *M*_int_ *=* 41,204; Figure 2C; Table 1), Power = 0.99.

The same analysis for the tasks with medium difficulty revealed a significant effect for the PT with *F*(2,55) *=* 100.22, *p <* 0.001 and *η^2^ =* 0.78. The post-hoc analysis supported the hypothesis and showed significant differences between *PT*_exp_: *PT*_nov_ (*M*_exp_ *=* 28,739: *M*_nov_ *=* 116,529), *PT*_exp_: *PT*_int_ (*M*_exp_ *=* 28,739: *M*_int_ *=* 77,162) and *PT*_int_: *PT*_nov_ (*M*_int_ *=* 77,162: *M*_nov_ *=* 116,529; Figure 2C; Table 1), Power = 1.00.

For the difficult tasks, the same analysis displays a significant effect for the PT with *F*(2,55) *=* 24.17, *p <* 0.001 and *η^2^ =* 0.47. The post-hoc analysis indicated significant differences between *PT*_exp_: *PT*_nov_ (*M*_exp_ *=* 99,200: *M*_nov_ *=* 134,128) and *PT*_exp_: *PT*_int_ (*M*_exp_ *=* 99,200: *M*_int_ *=* 130,625) but according to our hypothesis, no significant differences between *PT*_int_: *PT*_nov_ (*M*_int_ *=* 130,625: *M*_nov_ *=* 134,128; Figure 2C; Table 1), Power = 0.98.

#### Number of fixations

In the same line of reasoning as for PT, it was expected that experts’ and intermediates’ absolute number of fixations (*NF*) would be significantly lower than the NF of novices for easy and medium tasks as experts and intermediates should be able to identify relevant pieces/squares faster (Chase and Simon, 1973a,Chase and Simon, 1973b). For medium tasks, we also awaited that experts’ NF would be significantly lower than that of intermediates because intermediates would be more challenged by medium tasks than by easy tasks but experts would master both easy and faster. For difficult tasks, we anticipated the NF of experts to be significantly lower than the NF of novices and intermediates, because only experts would not be overstrained by difficult tasks. However, no significant differences between intermediates’ and novices’ NF were expected for difficult tasks as these are beyond the capabilities of both groups.

To test these hypotheses, one-way ANOVAs (condition, expertise) were performed for NF. For the easy tasks, the one-way ANOVA showed a significant effect for NF with *F*(2,55) *=* 45.00, *p <* 0.001 and *η^2^ =* 0.62. The post-hoc analysis revealed significant differences between *NF*_exp_: *NF*_nov_ (*M*_exp_ *=* 36.46: *M*_nov_ *=* 256.89) and *NF*_int_: *NF*_nov_ (*M*_int_ *=* 132.11: *M*_nov_ *=* 256.89), supporting the hypothesis, but unexpectedly also between *NF*_exp_: *NF*_int_ (*M*_exp_ *=* 36.46: *M*_int_ *=* 132.11; Figure 2A; Table 1), Power = 0.99.

The same analysis for the block of tasks with medium difficulty showed a significant effect for NF with *F*(2,55) *=* 61.84, *p <* 0.001, *η^2^ = *0.69. According to the hypothesis, the post-hoc comparison showed significant differences between *NF*_exp_: *NF*_nov_ (*M*_exp_ *=* 93.15: *M*_nov_ *=* 364.52), *NF*_exp_: *NF*_int_ (*M*_exp_ *=* 93.15: *M*_int_ *=* 236.50) and *NF*_int_: *NF*_nov_ (*M*_int_ *=* 236.50: *M*_nov_ *=* 364.52; Figure 2A; Table 1), Power = 0.99.

For the difficult tasks, the analogous analysis showed a significant effect for the NF with *F*(2,55) *=* 14.66, *p <* 0.001, *η^2^ =* 0.35. The Tukey LSD test indicated significant differences between *NF*_exp_: *NF*_nov_ (*M*_exp_ *=* 309.55: *M*_nov_ *=* 445.56) and *NF*_exp_: *NF*_int_ (*M*_exp_ *=* 309.55: *M*_int_ *=* 417.38), Power = 0.98, but no significant differences between *NF*_int_: *NF*_nov_ (*M*_int_ *=* 417.38: *M*_nov_ *=* 445.56; Figure 2A; Table 1) as hypothesised.

#### Duration of fixations

According to the results of the Reingold et al. (2001a) study, no significant expertise-dependent differences in fixation duration (*DF*) were anticipated. As the task difficulty increases, so does the total number of fixations and processing time. This progressive relationship probably impacts the visual searching strategies of all participants, resulting in no significant differences in DF between the three groups and for all experimental settings.

One-way ANOVAs (condition, expertise) were performed on DF and did not yield a significant result (Figure 2B; Table 1) as hypothesized. For the easy tasks, the one-way ANOVA showed *F*(2,55) *=* 0.19 and *p =* 0.82. For the block of tasks with medium difficulty the same analysis displayed *F*(2,55) *=* 2.30 and *p =* 0.11. Regarding the difficult tasks, the analogous analysis showed *F*(2,55) *=* 0.42 and *p =* 0.66.

### Variations in fixations on empty squares and on predetermined AOI

#### Number of fixations on empty squares

For complete trials, we have discussed the relative number of fixations on empty squares (*RNFES*). Based on the study by Reingold et al. (2001a) and Hainke and Pfeiffer (2021), we expected that experts’ RNFES between pieces would be significantly higher than novices’ and intermediates’ RNFES in all task blocks (easy, medium and difficult tasks). Following the same line of reasoning as for the RNFES, we anticipated that for the initial phase of task processing (the first 2, 3, 4 and 5 s), experts’ total number of fixations on empty squares (*NFES*) would be significantly higher than the NFES of novices and intermediates in all task blocks.

**Figure 3:**
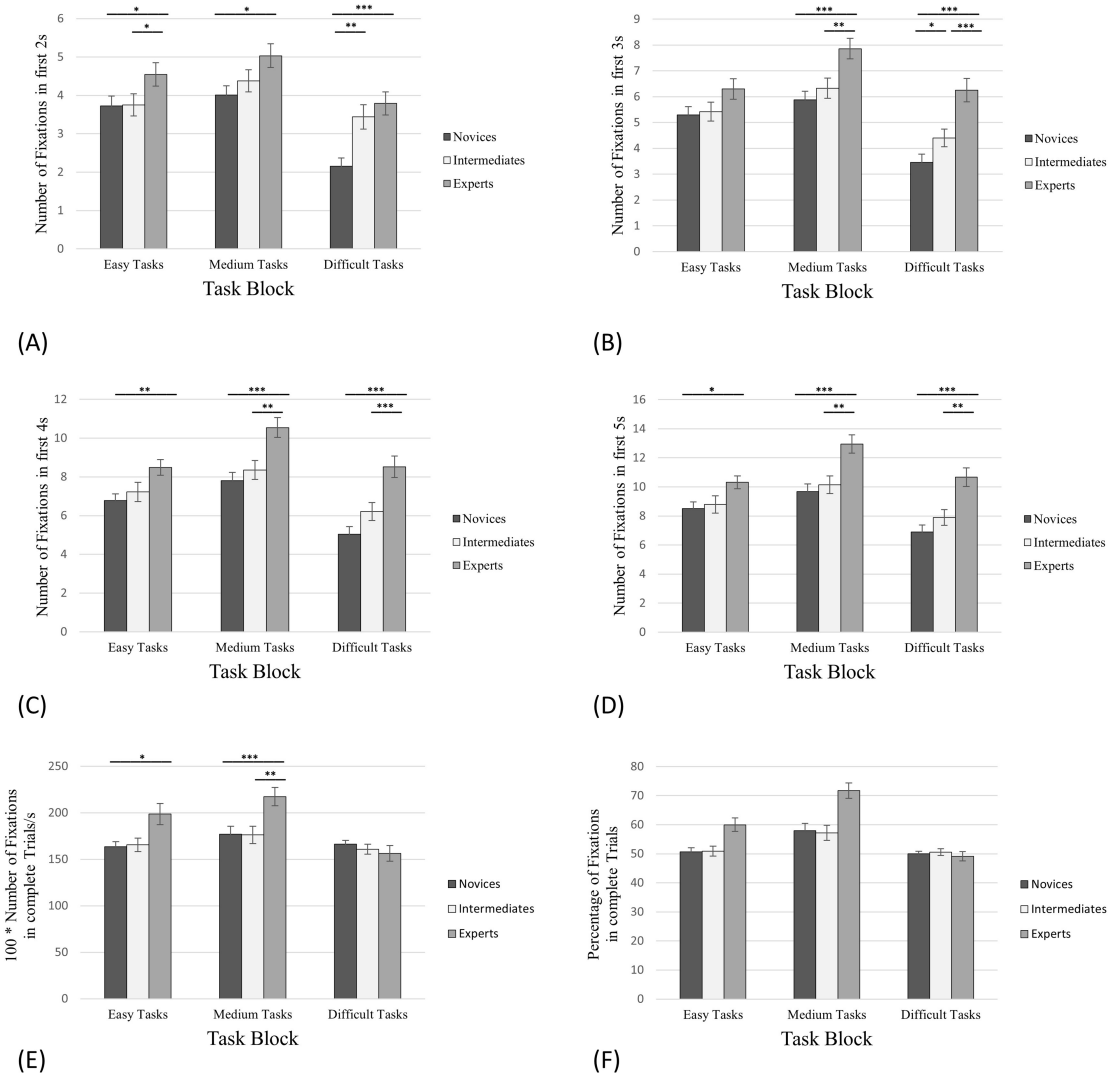
Number of fixations on empty squares between pieces. The error bars refer to the standard errors. **(A)** The first 2 s of task processing; **(B)** The first 3 s of task processing; **(C)** The first 4 s of task processing; **(D)** The first 5 s of task processing; **(E)** Complete trials (in this case in relation to the processing time); **(F)** The percentage of the total number of fixations on the corresponding AOIs in relation to all fixations on the board. * denotes “significantly” (i.e., *p* < 0.05). ** denotes “highly significant” (i.e., *p* < 0.01). *** denotes “extremely high significant” (i.e., *p* < 0.001).

Regarding complete trials, the results of the one-way ANOVA revealed a significant effect for the RNFES in case of easy tasks with *F*(2,55) = 3.44, *p* = 0.039, *η^2^* = 0.11. According to the hypothesis, the post-hoc analysis indicated significant differences between *RNFES*_exp_: *RNFES*_nov_ (*M*_exp_ *=* 193.57: *M*_nov_ *=* 163.63) but neither between *RNFES*_exp_: *RNFES*_int_ (*M*_exp_ *=* 193.57: *M*_int_ *=* 169.57) nor between *RNFES*_int_: *RNFES*_nov_ (*M*_int_ *=* 169.57: *M*_nov_ *=* 163.63; Figure 3E; Table 1), Power = 0.25.

The same analysis for the medium tasks showed a significant effect for the RNFES with *F*(2,55) = 9.51, *p* < 0.001, *η^2^* = 0.26. The post-hoc analysis revealed significant differences between *RNFES*_exp_: *RNFES*_nov_ (*M*_exp_ *=* 222.17: *M*_nov_ *=* 177.12), supporting the hypothesis, and unexpectedly between *RNFES*_exp_: *RNFES*_int_ (*M*_exp_ *=* 222.17: *M*_int_ *=* 177.20) but not between *RNFES*_int_: *RNFES*_nov_ (*M*_int_ *=* 177.20: *M*_nov_ *=* 177.12; Figure 3E; Table 1), Power = 0.40.

Contrary to the hypothesis, the one-way ANOVA showed no significant effect for the RNFES for the difficult tasks (Figure 3E; Table 1).

Moreover, we calculated the percentage of the total number of all fixations (*PFES*) on the board for complete trials that were only on empty squares (Figure 3C) as the PT of complete trials varies. In case of easy tasks, experts revealed 59.99% fixations on empty squares (SD = 18.99%, SE = 2.30%), intermediates 50.90% (SD = 14.94%, SE = 1.67%) and novices 50.68% (SD = 12.84%, SE = 1.40%). For the medium tasks, experts fixated 71.72% on empty squares (SD = 21.91%, SE = 2.66%), intermediates 57.22% (SD = 23.40%, SE = 2.62%) and novices 57.96% (SD = 22.46%, SE = 2.45%). The PFES in the difficult tasks was 49.11% for the experts (SD = 11.57%, SE = 1.62%), 50.56% for the intermediates (SD = 9.00%, SE = 1.16%) and 49.97% for the novices (SD = 7.38%, SE = 0.93%).

For almost all thresholds of the initial phase (Figures 3A–D), one-way ANOVAs (condition, expertise) for the NFES revealed significant between-group effects for all task blocks (easy, medium and difficult) (Tables 2–4). Only for the first 3 s of task processing of the easy tasks no significant effects could be indicated (Figure 3B). Exemplarily, we will only go into detail about the first 4 s of task processing (Figure 3C): Regarding the easy tasks the one-way ANOVA showed *F* (2,55) = 5.31, *p* = 0.008, *η^2^* = 0.16. The post-hoc analysis revealed significant differences between *NFES*_exp_: *NFES*_nov_ (*M*_exp_ *=* 8.44: *M*_nov_ *=* 6.77) but not between *NFES*_int_: *NFES*_nov_ (*M*_int_ *=* 7.19: *M*_nov_ *=* 6.77), Power = 0.23, supporting the hypothesis, and unexpectedly not between *NFES*_exp_: *NFES*_int_ (*M*_exp_ *=* 8.44: *M*_int_ *=* 7.19; Figure 3C; Table 2).

**Table 2:** Results of one-way ANOVAs with special consideration of thresholds for the initial phase of task processing (easy tasks).

		Group comparison (ANOVAs and Bonferroni multiple comparison post-hoc tests) for the easy tasks (1 = Novices; 2 = Intermediates; 3 = Experts)			
	Time threshold [s]	ANOVA (main effect) F, *p* and η^2^ values	Post-hoc comparisons 1–2	Post-hoc comparisons 1–3	Post-hoc comparisons 2–3
Empty squares	2	*F* = 5.680, *p* = 0.006, η^2^ = 0.17	*p* = 1.000	*p* = 0.012^*^ (*M*_exp_ *=* 4.59: *M*_nov_ *=* 3.64)	*p* = 0.015^*^ (*M*_exp_ *=* 4.59: *M*_int_ *=* 3.66)
	3	*F* = 2.620, *p* = 0.082, η^2^ = 0.09	*p* = 1.000	*p* = 0.118	*p* = 0.189
	4	*F* = 5.310, *p* = 0.008, η^2^ = 0.16	*p* = 1.000	*p* = 0.008^**^ (*M*_exp_ = 8.44: *M*_nov_ *=* 6.77)	*p* = 0.070
	5	*F* = 4.000, *p* = 0.026, η^2^ = 0.13	*p* = 1.000	*p* = 0.033^*^ (*M*_exp_ = 10.32: *M*_nov_ = 8.51)	*p* = 0.076
AOI 1	2	*F* = 0.750, *p* = 0.476, η^2^ = 0.03	*p* = 0.689	*p* = 1.000	*p* = 1.000
	3	*F* = 0.370, *p* = 0.695, η^2^ = 0.01	*p* = 1.000	*p* = 1.000	*p* = 1.000
	4	*F* = 0.900, *p* = 0.412, η^2^ = 0.03	*p* = 0.601	*p* = 1.000	*p* = 1.000
	5	*F* = 1.210, *p* = 0.307, η^2^ = 0.04	*p* = 0.413	*p* = 0.910	*p* = 1.000
AOI 2	2	*F* = 0.140, *p* = 0.867, η^2^ = 0.01	*p* = 1.000	*p* = 1.000	*p* = 1.000
	3	*F* = 0.050, *p* = 0.954, η^2^ < 0.01	*p* = 1.000	*p* = 1.000	*p* = 1.000
	4	*F* = 0.010, *p* = 0.986, η^2^ < 0.01	*p* = 1.000	*p* = 1.000	*p* = 1.000
	5	*F* = 0.160, *p* = 0.856, η^2^ = 0.01	*p* = 1.000	*p* = 1.000	*p* = 1.000
AOI 3	2	*F* = 0.640, *p* = 0.532, η^2^ = 0.02	*p* = 1.000	*p* = 0.814	*p* = 1.000
	3	*F* = 0.390, *p* = 0.688, η^2^ = 0.01	*p* = 1.000	*p* = 1.000	*p* = 1.000
	4	*F* = 0.440, *p* = 0.645, η^2^ = 0.02	*p* = 1.000	*p* = 1.000	*p* = 1.000
	5	*F* = 0.280, *p* = 0.759, η^2^ = 0.01	*p* = 1.000	*p* = 1.000	*p* = 1.000

* denotes “significantly” (i.e., *p* < 0.05). ** denotes “highly significant” (i.e., *p* < 0.01). *** denotes “extremely high significant” (i.e., *p* < 0.001).

**Table 3:** Results of one-way ANOVAs with special consideration of thresholds for the initial phase of task processing (medium tasks).

		Group comparison (ANOVAs and Bonferroni multiple comparison post-hoc tests) for the medium tasks (1 = Novices; 2 = Intermediates; 3 = Experts)			
	Time threshold [s]	ANOVA (main effect) F, *p* and η^2^ values	Post-hoc comparisons 1–2	Post-hoc comparisons 1–3	Post-hoc comparisons 2–3
Empty squares	2	*F* = 4.010, *p* = 0.024, η^2^ = 0.13	*p* = 0.896 (*M*_int_ *=* 4.38)	*p* = 0.020^*^ (*M*_exp_ = 5.03: *M*_nov_ = 4.01)	*p* = 0.236
	3	*F* = 9.880, *p* < 0.001, η^2^ = 0.26	*p* = 0.918	*p* < 0.001^***^ (*M*_exp_ = 7.87: *M*_nov_ = 5.88)	*p* = 0.005^**^ (*M*_exp_ = 7.87: *M*_int_ = 6.34)
	4	*F* = 12.180, *p* < 0.001, η^2^ = 0.31	*p* = 0.888	*p* < 0.001^***^ (*M*_exp_ = 10.47: *M*_nov_ = 7.81)	*p* = 0.001^**^ (*M*_exp_ = 10.47: *M*_int_ = 8.38)
	5	*F* = 11.680, *p* < 0.001, η^2^ = 0.30	*p* = 1.000	*p* < 0.001^***^ (*M*_exp_ = 12.96: *M*_nov_ = 9.68)	*p* = 0.001^**^ (*M*_exp_ = 12.96: *M*_int_ = 10.14)
AOI 1	2	*F* = 3.610, *p* = 0.034, η^2^ = 0.12	*p* = 1.000 (*M*_int_ *=* 1.26)	*p* = 0.031^*^ (*M*_exp_ *=* 1.74: *M*_nov_ *=* 1.05)	*p* = 0.230
	3	*F* = 5.190, *p* = 0.009, η^2^ = 0.16	*p* = 0.623 (*M*_int_ *=* 1.89)	*p* = 0.007^**^ (*M*_exp_ *=* 2.50: *M*_nov_ *=* 1.51)	*p* = 0.162
	4	*F* = 8.160, *p* < 0.001, η^2^ = 0.23	*p* = 0.126 (*M*_int_ *=* 2.63)	*p* = 0.001^***^ (*M*_exp_ *=* 3.34: *M*_nov_ *=* 1.93)	*p* = 0.145
	5	*F* = 5.730, *p* = 0.005, η^2^ = 0.17	*p* = 0.431 (*M*_int_ *=* 3.18)	*p* = 0.004^**^ (*M*_exp_ *=* 3.93: *M*_nov_ *=* 2.63)	*p* = 0.172
AOI 2	2	*F* = 7.570, *p* = 0.001, η^2^ = 0.22	*p* = 0.087 (*M*_int_ *=* 2.55)	*p* = 0.001^***^ (*M*_exp_ *=* 3.13: *M*_nov_ *=* 1.82)	*p* = 0.287
	3	*F* = 10.740, *p* < 0.001, η^2^ = 0.28	*p* = 0.069, (*M*_int_ *=* 3.84)	*p* = 0.001^***^ (*M*_exp_ *=* 4.90: *M*_nov_ *=* 2.85)	*p* = 0.065
	4	*F* = 9.920, *p* < 0.001, η^2^ = 0.27	*p* = 0.067 (*M*_int_ *=* 5.26)	*p* = 0.001^***^, (*M*_exp_ *=* 6.56: *M*_nov_ *=* 3.93)	*p* = 0.104
	5	*F* = 9.100, *p* < 0.001, η^2^ = 0.25	*p* = 0.074 (*M*_int_ *=* 6.80)	*p* = 0.001^***^ (*M*_exp_ *=* 8.29: *M*_nov_ *=* 5.18)	*p* = 0.146
AOI 3	2	*F* = 5.130, *p* = 0.009, η^2^ = 0.16	*p* = 0.225 (*M*_int_ *=* 4.58)	*p* = 0.007^**^ (*M*_exp_ *=* 5.22: *M*_nov_ *=* 3.80)	*p* = 0.477
	3	*F* = 7.130, *p* = 0.002, η^2^ = 0.21	*p* = 0.143 (*M*_int_ *=* 7.03)	*p* = 0.001^**^ (*M*_exp_ *=* 8.15: *M*_nov_ *=* 5.83)	*p* = 0.230
	4	*F* = 6.240, *p* = 0.004, η^2^ = 0.18	*p* = 0.172 (*M*_int_ *=* 9.53)	*p* = 0.003^**^ (*M*_exp_ *=* 10.74: *M*_nov_ *=* 8.17)	*p* = 0.321
	5	*F* = 7.560, *p* = 0.001, η^2^ = 0.22	*p* = 0.210 (*M*_int_ *=* 11.81)	*p* = 0.001^***^ (*M*_exp_ *=* 13.50: *M*_nov_ *=* 10.41)	*p* = 0.122

* denotes “significantly” (i.e., *p* < 0.05). ** denotes “highly significant” (i.e., *p* < 0.01). *** denotes “extremely high significant” (i.e., *p* < 0.001).

**Table 4:** Results of one-way ANOVAs with special consideration of thresholds for the initial phase of task processing (difficult tasks).

		Group comparison (ANOVAs and Bonferroni multiple comparison post-hoc tests) for the difficult tasks (1 = Novices; 2 = Intermediates; 3 = Experts)			
	Time threshold [s]	ANOVA (main effect) F, *p* and η^2^ values	Post-hoc comparisons 1–2	Post-hoc comparisons 1–3	Post-hoc comparisons 2–3
Empty squares	2	*F* = 11.970, *p* < 0.001, η^2^ = 0.30	*p* = 0.002^**^ (*M*_int_ *=* 3.40: *M*_nov_ *=* 2.13)	*p* < 0.001^***^ (*M*_exp_ = 3.78: *M*_nov_ = 2.13)	*p* = 0.896
	3	*F* = 28.630, *p* < 0.001, η^2^ = 0.51	*p* = 0.008^*^ (*M*_int_ *=* 4.47: *M*_nov_ *=* 3.29)	*p* < 0.001^***^ (*M*_exp_ = 6.26: *M*_nov_ = 3.29)	*p* < 0.001^***^ (*M*_exp_ = 6.26: *M*_int_ = 4.47)
	4	*F* = 17.920, *p* < 0.001, η^2^ = 0.39	*p* = 0.121	*p* < 0.001^***^ (*M*_exp_ = 8.41: *M*_nov_ = 5.02)	*p* < 0.001^***^ (*M*_exp_ = 8.41: *M*_int_ = 6.17)
	5	*F* = 14.310, *p* < 0.001, η^2^ = 0.34	*p* = 0.298	*p* < 0.001^***^ (*M*_exp_ = 10.67: *M*_nov_ = 6.86)	*p* = 0.002^**^ (*M*_exp_ = 10.67: *M*_int_ = 8.02)
AOI 1	2	*F* = 0.430, *p* = 0.653, η^2^ = 0.02	*p* = 1.000	*p* = 1.000	*p* = 1.000
	3	*F* = 0.100, *p* = 0.903, η^2^ < 0.01	*p* = 1.000	*p* = 1.000	*p* = 1.000
	4	*F* = 0.790, *p* = 0.457, η^2^ = 0.03	*p* = 1.00	*p* = 0.654	*p* = 1.000
	5	*F* = 1.170, *p* = 0.317, η^2^ = 0.04	*p* = 0.911	*p* = 0.433	*p* = 1.000
AOI 2	2	F = 0.160, *p* = 0.852, η^2^ = 0.01	*p* = 1.000	*p* = 1.000	*p* = 1.000
	3	*F* = 0.300, *p* = 0.739, η^2^ = 0.01	*p* = 1.000	*p* = 1.000	*p* = 1.000
	4	*F* = 0.730, *p* = 0.486, η^2^ = 0.03	*p* = 1.000	*p* = 1.000	*p* = 0.731
	5	*F* = 0.980, *p* = 0.380, η^2^ = 0.03	*p* = 1.000	*p* = 0.693	*p* = 0.645
AOI 3	2	*F* = 3.960, *p* = 0.025, η^2^ = 0.13	*p* = 1.000 (*M*_int_ *=* 4.57)	*p* = 0.027^*^ (*M*_exp_ *=* 5.69: *M*_nov_ *=* 4.27)	*p* = 0.110
	3	*F* = 5.660, *p* = 0.006, η^2^ = 0.17	*p* = 1.000	*p* = 0.009^**^ (*M*_exp_ *=* 8.86: *M*_nov_ *=* 6.51)	*p* = 0.021^*^ (*M*_exp_ *=* 8.86: *M*_int_ *=* 6.70)
	4	*F* = 7.070, *p* = 0.002, η^2^ = 0.20	*p* = 1.000	*p* = 0.002^**^(*M*_exp_ *=* 12.08: *M*_nov_ *=* 8.76)	*p* = 0.017^*^ (*M*_exp_ *=* 12.08: *M*_int_ *=* 9.38)
	5	*F* = 6.630, *p* = 0.003, η^2^ = 0.19	*p* = 1.000	*p* = 0.003^**^ (*M*_exp_ *=* 15.17: *M*_nov_ *=* 11.51)	*p* = 0.016^*^ (*M*_exp_ *=* 15.17: *M*_int_ *=* 12.05)

* denotes “significantly” (i.e., *p* < 0.05). ** denotes “highly significant” (i.e., *p* < 0.01). *** denotes “extremely high significant” (i.e., *p* < 0.001).

Regarding the medium tasks, the results of the one-way ANOVA are *F*(2,55) = 12.18, *p* < 0.001, *η^2^* = 0.31. The post-hoc analysis revealed significant differences between *NFES*_exp_: *NFES*_nov_ (*M*_exp_ *=* 10.47: *M*_nov_ *=* 7.81) and *NFES*_exp_: *NFES*_int_ (*M*_exp_ *=* 10.47: *M*_int_ *=* 8.38), Power = 0.43, but no significant differences between *NFES*_int_: *NFES*_nov_ (*M*_int_ *=* 8.38: *M*_nov_ *=* 7.81; Figure 3C; Table 3), supporting the hypothesis.

The same analysis for the difficult tasks revealed a significant effect for the NFES, *F*(2,55) = 17.92, *p* < 0.001, *η^2^* = 0.39. The post-hoc analysis showed significant differences between *NFES*_exp_: *NFES*_nov_ (*M*_exp_ *=* 8.41: *M*_nov_ *=* 5.02) and *NFES*_exp_: *NFES*_int_ (*M*_exp_ *=* 8.41: *M*_int_ *=* 6.17), Power = 0.76, but not between *NFES*_int_: *NFES*_nov_ (*M*_int_ *=* 6.17: *M*_nov_ *=* 5.02; Figure 3C; Table 4), confirming the hypothesis.

#### Number of fixations on predetermined AOI

##### Number of fixations on AOI 1

Considering the complete trials, we did not anticipate significant between-group differences in the relative number of fixations on AOI 1 (*RNFAOI 1*) for the easy tasks, as they contain elementary attacks familiar to all expertise groups. However, concerning the medium and difficult tasks, we awaited experts’ *RNFAOI 1* to be significantly higher than novices’ and intermediates’ *RNFAOI 1.* We argue that both medium and difficult tasks would be more demanding in terms of identifying relevant pieces. Especially for the difficult tasks, we did not anticipate any significant differences between the *RNFAOI 1* of intermediates and novices, as we assume that the difficult tasks are almost equally stressful for both groups.

**Figure 4:**
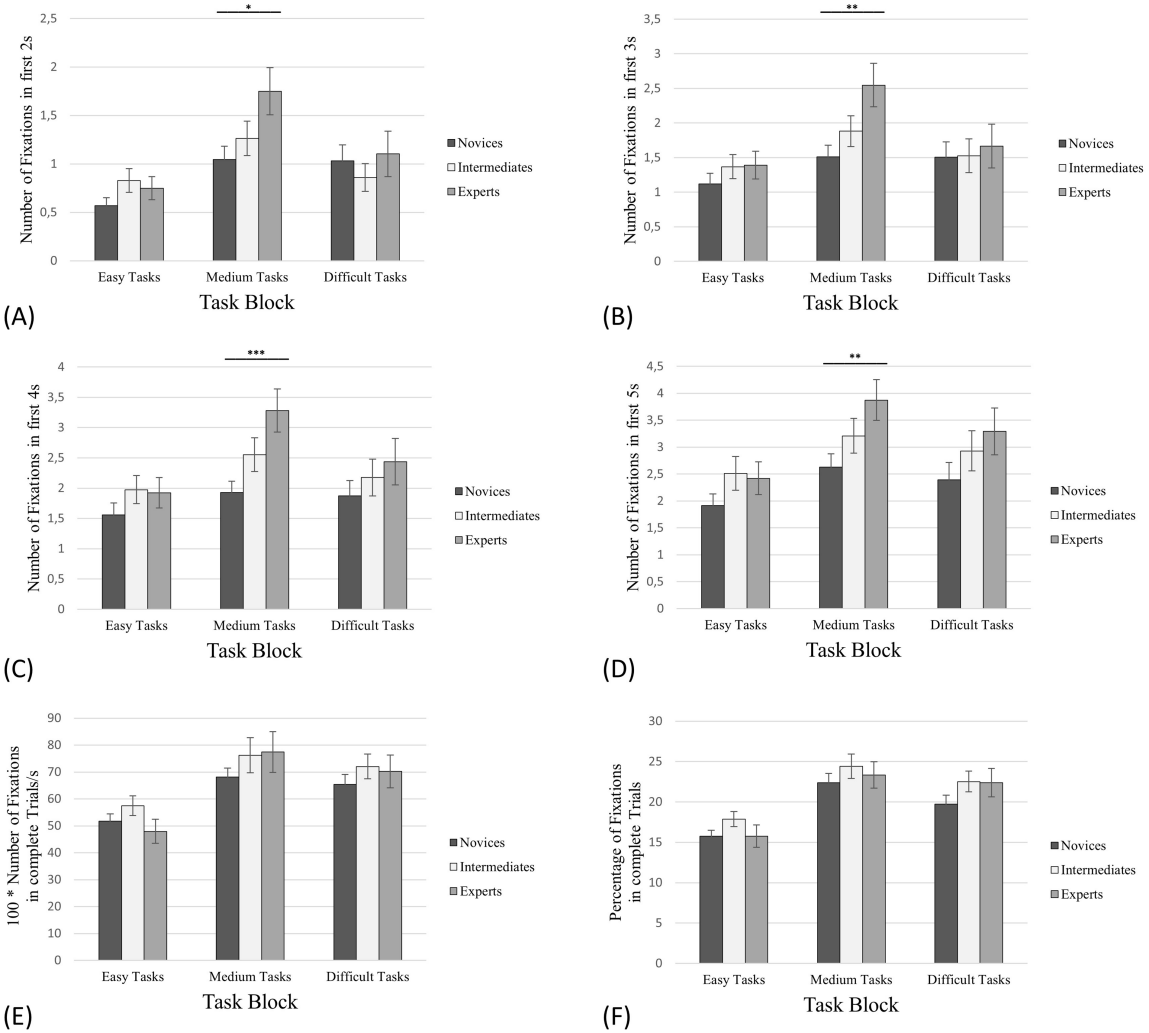
Number of fixations on AOI 1. The error bars refer to the standard errors. **(A)** The first 2 s of task processing; **(B)** The first 3 s of task processing; **(C)** The first 4 s of task processing; **(D)** The first 5 s of task processing; **(E)** Complete trials (in this case in relation to the processing time); **(F)** The percentage of the total number of fixations on the corresponding AOIs in relation to all fixations on the board. * denotes “significantly” (i.e., *p* < 0.05). ** denotes “highly significant” (i.e., *p* < 0.01). *** denotes “extremely high significant” (i.e., *p* < 0.001).

One-way ANOVAs (condition, expertise) unexpectedly revealed no significant between-group effects for RNFAOI 1 for any task block (Figure 4E; Table 1).

In the same way as for PFES, we also calculated and displayed the percentage of the total number of all fixations (complete trials) on the board that were on AOI 1 only (*PFAOI 1*—Figure 4F). For the easy tasks, experts fixated 15.75% on AOI 1 (SD = 11.43%, SE = 1.39%), intermediates 17.88% (SD = 8.40%, SE = 0.94%) and novices 15.75% (SD = 6.77%, SE = 0.74%). The PFAOI 1 of experts for the medium tasks was 23.35% (SD = 13.41%, SE = 1.63%), for intermediates 24.42% (SD = 13.49%, SE = 1.51%) and for novices 22.39% (SD = 10.49%, SE = 1.14%). In case of the difficult tasks, experts revealed 22.38% fixations on AOI 1 (SD = 12.48%, SE = 1.75%), intermediates 22.53% (SD = 9.91%, SE = 1.28%) and novices 19.74% (SD = 8.63%, SE = 1.09%).

Concerning the initial phase of the easy tasks, we expected both experts’ and intermediates’ NFAOI 1 to be significantly higher than novices’ NFAOI 1, because we assumed that only novices would not be able to identify relevant pieces from the beginning, but that experts and intermediates would perform similarly well in this early phase of task processing. On the basis of the study by Chase and Simon (1973a,b), we anticipated that the NFAOI 1 of the experts would be significantly higher than the NFAOI 1 of the novices for the medium tasks and that no further effects would be indicated. We argue that identifying relevant pieces within the first 5 s may be challenging for both novices and intermediates, but intermediates would have fewer problems than novices. We did not anticipate this effect to cause any significant differences between intermediates and experts, nor between intermediates and novices. Regarding the difficult tasks, we expected that the experts’ NFAOI 1 would be significantly higher than the NFAOI 1 of intermediates and novices, as only the experts may be able to identify relevant pieces within the first 2–5 s.

One-way ANOVAs (condition, expertise) for NFAOI 1 in the initial phase unexpectedly revealed significant between-group effects only for the medium tasks (Figures 4A–D; Tables 2–4). This supports the hypothesis for the medium tasks only.

##### Number of fixations on AOI 2

Compared to AOI 1, the aspect of empty squares comes along in AOI 2. We hypothesise that the differences in *NFES* described above (Figure 3E; Table 1) would induce those between-group effects which could not be indicated for RNFAOI 1 (Figure 4E; Table 1), as AOI 1 does not contain any piece replacement squares except for the destination square(s) of an attacker/ of attackers, as described in the section above. Therefore, we expected that the relative number of fixations on AOI 2 (*RNFAOI 2*) of experts would be significantly higher than the RNFAOI 2 of novices and intermediates for medium and difficult tasks in complete trials. In the same line of reasoning, we anticipated similar and more significant results for the total number of fixations on AOI 2 (*NFAOI 2*) in the initial phase compared to *NFAOI 1* in the medium and difficult tasks.

**Figure 5:**
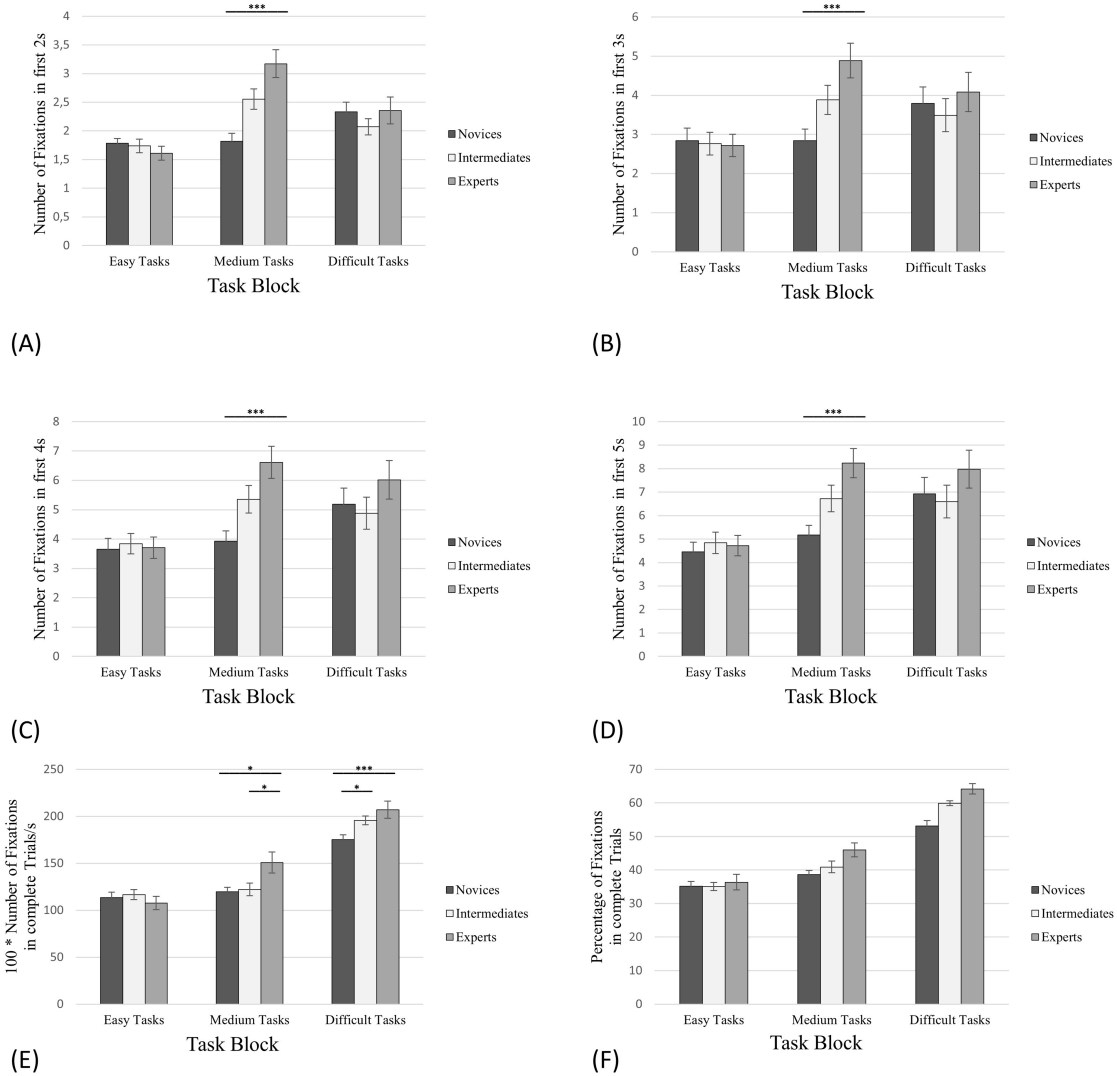
Number of fixations on AOI 2. The error bars refer to the standard errors. **(A)** The first 2s of task processing; **(B)** The first 3 s of task processing; **(C)** The first 4 s of task processing; **(D)** The first 5 s of task processing; **(E)** Complete trials (in this case in relation to the processing time); **(F)** The percentage of the total number of fixations on the corresponding AOIs in relation to all fixations on the board. * denotes “significantly” (i.e., *p* < 0.05). ** denotes “highly significant” (i.e., *p* < 0.01). *** denotes “extremely high significant” (i.e., *p* < 0.001).

In contrast to RNFAOI 1, one-way ANOVAs (condition, expertise) for RNFAOI 2 in complete trials revealed significant between-group effects for medium and difficult tasks: Concerning the medium tasks, the one-way ANOVA showed a significant effect for the RNFAOI 2 with *F*(2,55) = 4.88, *p* = 0.011, *η^2^* = 0.15. The post-hoc analysis revealed significant differences between *RNFAOI 2*_exp_: *RNFAOI 2*_nov_ (*M*_exp_ *=* 154.23: *M*_nov_ *=* 119.70) and *RNFAOI 2*_exp_: *RNFAOI 2*_int_ (*M*_exp_ *=* 154.23: *M*_int_ *=* 122.89), Power = 0.31, but not for *RNFAOI 2*_int_: *RNFAOI 2*_nov_ (*M*_int_ *=* 122.89: *M*_nov_ *=* 119.70; Figure 5E; Table 1), supporting the hypothesis.

For the difficult tasks, the results of the one-way ANOVA indicated a significant effect for the RNFAOI 2 with *F*(2,55) = 7.88, *p* < 0.001, *η^2^* = 0.22, Power = 0.66. According to the hypothesis, the post-hoc analysis showed significant differences between *RNFAOI 2*_exp_: *RNFAOI 2*_nov_ (*M*_exp_ *=* 213.22: *M*_nov_ *=* 175.19) but unexpectedly also between *RNFAOI 2*_int_: *RNAOI 2*_nov_ (*M*_int_ *=* 199.84: *M*_nov_ = 175.19) and not between *RNFAOI 2*_exp_: *RNFAOI 2*_int_ (*M*_exp_ *=* 213.22: *M*_int_ *=* 199.84; Figure 5E; Table 1). In the same line of reasoning as for PFES and PFAOI 1, we calculated and displayed how many percent of the total number of all fixations (complete trials) on the board were on AOI 2 only (*PFAOI 2*) (Figure 5F). The PFAOI 2 of experts for the easy tasks was 36.36% (SD = 19.26%, SE = 2.34%), for intermediates 35.08% (SD = 10.89%, SE = 1.22%) and for novices 35.14% (SD = 13.50%, SE = 1.47%). In case of the medium tasks, experts revealed 45.99% fixations on AOI 2 (SD = 17.13%, SE = 2.08%), intermediates 40.90% (SD = 15.47%, SE = 1.73%) and novices 38.62% (SD = 11.71%, SE = 1,28%). For the difficult tasks, experts showed 64.18% fixations on AOI 2 (SD = 11.14%, SE = 1.56%), intermediates 59.90% (SD = 5.82%, SE = 0.75%) and novices 53.14% (SD = 12.50%, SE = 1.57%).

Finally, concerning the initial phase, one-way ANOVAs (condition, expertise) for NFAOI 2 revealed significant between-group effects only for the medium tasks (Figures 5A–D; Tables 2–4): This supports the hypothesis only for the medium tasks.

##### Number of fixations on AOI 3

For full trials and the initial phase of task processing, we expected similar but even more significant between-group differences for the relative number of fixations on AOI 3 (*RNFAOI 3*) compared to RNFAOI 2 and for the total number of fixations on AOI 3 (*NFAOI 3*) compared to NFAOI 2. We argued that fixations outside of AOI 3 clearly indicate a lack of understanding which we attributed to novices at least for medium and difficult tasks. On the contrary, the fixations on AOI 3 indicate at least a rudimentary understanding of the task which we assigned to the intermediates in terms of medium and difficult tasks. Consequently, we awaited that for all task blocks, experts’ RNFAOI 3 and NFAOI 3 would be significantly higher than the RNFAOI 3 and NFAOI 3 of the other groups, and the RNFAOI 3 and NFAOI 3 of the intermediates would be significantly higher than the RNFAOI 3 and NFAOI 3 of the novices.

**Figure 6:**
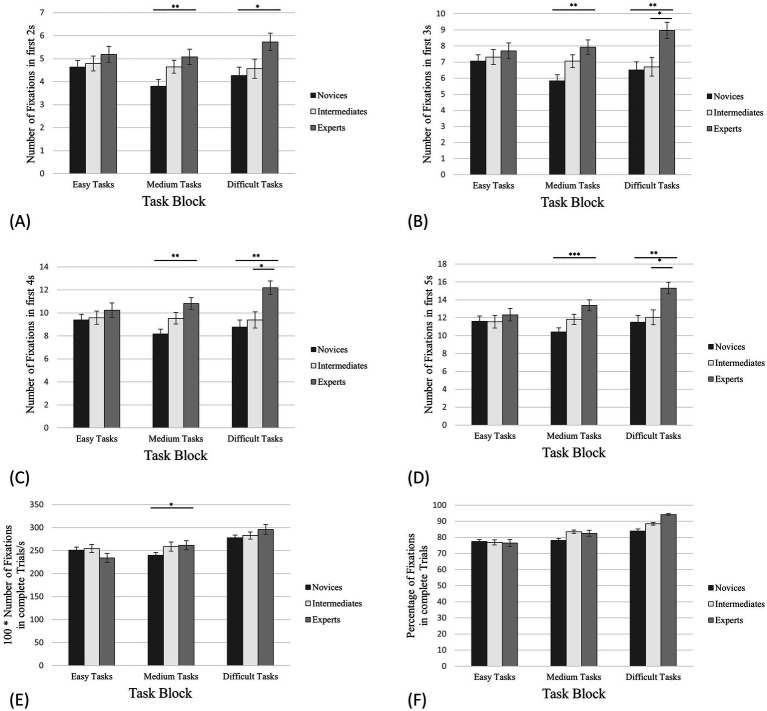
Number of fixations on AOI 3. The error bars refer to the standard errors. **(A)** The first 2 s of task processing; **(B)** The first 3 s of task processing; **(C)** The first 4 s of task processing; **(D)** The first 5 s of task processing; **(E)** Complete trials (in this case in relation to the processing time); **(F)** The percentage of the total number of fixations on the corresponding AOIs in relation to all fixations on the board. * denotes “significantly” (i.e., *p* < 0.05). ** denotes “highly significant” (i.e., *p* < 0.01). *** denotes “extremely high significant” (i.e., *p* < 0.001).

In the case of full trials, one-way ANOVAs for RNFAOI 3 showed significant between-group effects only for medium tasks: The results of the one-way ANOVA revealed a significant effect for the RNFAOI 3 with *F*(2,55) =3.36, *p* = 0.042, *η^2^* = 0.11, Power = 0.19. The post-hoc analysis indicated significant differences between *RNFAOI 3*_exp_: *RNFAOI 3*_nov_ (*M*_exp_ *=* 269.79: *M*_nov_ *=* 239.67) but neither between *RNFAOI 3*_exp_: *RNFAOI 3*_int_ (*M*_exp_ *=* 269.79: *M*_int_ *=* 261.28) nor between *RNFAOI 3*_int_: *RNAOI 3*_nov_ (*M*_int_ *=* 261.28: *M*_nov_ *=* 239.67; Figure 6E; Table 1), only supporting a small part of the hypothesis.

In the same line of reasoning as for PFES, PFAOI 1 and PFAOI 2, we have displayed how many percent of the total number of all fixations on the board were only on AOI 3 (*PFAOI 3*; Figure 6F). In the case of easy tasks, experts’ rate of fixations on AOI 3 was 76.46% (SD = 17.30%, SE = 2.10%), intermediates showed 76.90% (SD = 14.45%, SE = 1.62%) and novices 77.46% of fixations on AOI 3 (SD = 11.21%, SE = 1.22%). For the medium tasks, experts fixated 82.58% on AOI 3 (SD = 15.37%, SE = 1.86%), intermediates 83.53% (SD = 8.78%, SE = 0.98%) and novices 78.05% (SD = 11.07%, SE = 1.21%). The PFAOI 3 in the difficult tasks was 94.18% for the experts (SD = 3.78%, SE = 0.53%), 88.40% for the intermediates (SD = 6.11%, SE = 0.79%) and 83.97% for the novices (SD = 10.17%, SE = 1.28%). For the initial phase, one-way ANOVAs (condition, expertise) for NFAOI 3 showed the same significant between-group effects for the medium tasks (Figures 6A–D; Table 3) as for NFAOI 1 and NFAOI 2. However, in contrast to NFAOI 1 and NFAOI 2, significant between-group effects were also indicated for the difficult tasks (Figures 6A–D; Table 4).

## Discussion

High-level performance in chess is linked with highly developed visuocognitive skills (e.g., Charness et al., 2001), which facilitate the processing of visual information and are associated with experience and intensive practice/ dedicated practice (e.g., Ackerman and Cianciolo, 2000). Experts demonstrate these skills by applying efficient visual search strategies with fewer fixations (Reingold et al., 2001a; Reingold and Charness, 2005) and by quickly determining relevant regions in problem-solving tasks (Sheridan and Reingold, 2014). They also show faster identification of relevant move sequences (Sheridan and Reingold, 2017) as well as faster and more accurate decision-making and subliminal response priming effects (Kiesel et al., 2009; Postal, 2012; Küchelmann et al., 2022).

We used eye tracking in a real game scenario, automatic movement-time registration and data annotation (Küchelmann et al., 2017). The main effort of the present study was to extend existing findings through a decisive task manipulation, a circumstantial differentiation of expertise, an increase in the relevant dependent variables and a differentiated analysis of gaze behaviour according to both space and time. Concerning space, we employed ascending sequences of AOIs. Concerning time, we took into account that n-mate-tasks in case of n > 1 require a much more complex movement-planning than find-the-best-move tasks (e.g., in the study of Charness et al., 2001; Sheridan and Reingold, 2014), so we analysed both complete trials and a fine-graded sequence of several time intervals (in contrast to Hainke and Pfeiffer (2021) who chose only the cut off interval of the first 5 s) for the beginning phase of task processing (information peak).

Firstly, we hypothesised that experts would respond significantly more accurately than the other two groups and that the intermediates would perform better than novices in all tasks. The results confirmed our hypothesis. For the experts-novices differences, we consider this to be in line with the results of the Chase and Simon (1973a,b) studies. According to the literature the results can be explained by the expertise-dependent visuocognitive skills which lead to a more accurate decision-making.

Second, we hypothesised that experts would show a significantly lower processing time and absolute number of fixations on the board than novices for all tasks. We expected to find differences between experts and intermediates only for medium and difficult tasks and that intermediates-novices differences would only occur in case of easy and medium tasks. Statistical analysis supports this hypothesis in most instances. The statistical results confirm the findings of Reingold et al. (2001a) that the encoding of chunks results in fewer fixations and indicate that the task difficulty is a distinguishing feature in terms of visuocognitive performance between the participating groups.

Thirdly, we expected that there would be no significant expertise-dependent differences in fixation durations. The results confirmed our hypothesis and are consistent with the results of Reingold et al. (2001a).

A further analysis taking into account the total amount of fixations on empty squares (normalized by processing time) showed that experts had a significantly higher focus on the empty squares between the relevant pieces, supporting our hypothesis and the findings of Reingold et al. (2001a) and Hainke and Pfeiffer (2021) that the encoding of chunks results in a high proportion of fixations between rather than on pieces. For easy and medium tasks, experts reveal different visual search strategies, which partially supports our hypothesis. Regarding the medium tasks, experts had significantly more fixations on empty squares than the other groups, but for the easy tasks only experts-novices differences could be illustrated. This could be explained by the fact that intermediates used visual search strategies more similar to those of experts, but only for the easy task condition. Regarding the difficult tasks, no significant differences could be found between the three groups. The requirements of the difficult tasks force all participants to pre-arrange all movements needed (mate in four to six moves). This could lead the participants of all groups to an intensive search of the board and may serve to impose a limit on the selection of tasks with appropriate level of complexity in the context of eye tracking. As a result, a similarity in the fixations on the empty squares between the three groups with respect to the complete trials would be revealed. However, this analysis (number of fixations on empty squares for complete trials) does not allow us to determine the differences between the groups regarding the information peak in the initial phase of task processing [e.g. the analysis of the first five fixations per trial in Sheridan and Reingold, 2014]. This is why, in a second step, we carry out the detailed and comprehensive analysis of the differences concerning both spatial and temporal high resolution.

In order to achieve a visuo-spatial differentiation and to take into account complete trials of all conditions, we employed three gradually expanding AOIs: AOI 1 includes only the immediately relevant attacking pieces, the attackers’ target squares and the attacked king. AOI 2 provides a more dynamic perspective as it combines AOI 1 with the piece replacement squares (mainly empty squares) that reflect the trajectories and the planning of the attacking move(s). Finally, AOI 3 contains AOI 2 and all the nearest neighbour squares of the AOI 1 squares. This is because no region outside of AOI 3 contributes to the understanding of the task and fixations on AOI 3 should indicate at least a basic understanding of the task.

We hypothesised that the larger the areas of interest, the more likely it would be that expertise-dependent differences reflect the visuocognitive superiority of experts (e.g., chunks). The differentiated analyses showed no significant between-group effects for AOI 1. We argue that for the complete processing, merely identifying the relevant pieces is not the essential skill for solving the task. Hence, the attacked king – an extremely important element of AOI 1 – is a relevant piece in *every* task and therefore should be identified by *all* players, regardless of their expertise. For AOI 2, the results indicated experts-novices and experts-intermediates differences for medium tasks and expert-novices and intermediates-novices differences for difficult tasks. We argue that the aspect of empty squares between pieces plays a role in AOI 2, as they are essential for planning attacking moves. Furthermore, we suggest that information processing of novices, in contrast to the other two groups, may have been distracted by the cognitive overload caused by the difficulty of the task. For AOI 3, the statistical analysis only indicated a significant difference between experts and novices, and only for medium tasks.

In an attempt to itemise the amount and the quality of the information peak during the trials and the differences between the groups under consideration of the different conditions, a fine-graded temporal analysis with time intervals of increasing length was carried out. It has shown that, at least for medium tasks, and in case of AOI 3 also for difficult tasks, experts show a significantly higher number of fixations on empty squares as well as on all AOIs in the initial phase which is in line with results of Charness et al. (2001). Within the first 2 s, the experts-novices differences for fixation on empty squares are significant for all tasks (Figure 3A) – hence, this was not the case for complete trials and difficult tasks (Figure 3E). For fixations on AOI 1 and AOI 2, this is only true for medium tasks, and for AOI 3, experts-novices differences occur for both medium as difficult tasks. Within the first 3 s, experts-novices and experts-intermediates differences were only indicated for fixations on empty squares in medium and difficult tasks, the latter also showing differences between intermediates and novices. For fixations on AOI 1 and AOI 2, the revealed effects are similar to those for 2 s, but additional differences between experts and intermediates are indicated for AOI 3 and difficult tasks. However, at this point we have to express our concerns regarding the *post hoc* power analysis which is very low. We assume that this power value can be associated with the low number of trials. With respect to the first 4 s, the results showed the same between-group effects for fixations on AOI 1 and AOI 2 as the data reveal for the first 2 s. For fixations on empty squares, significant differences were found between experts and novices for all tasks, and between experts and novices only for the difficult tasks. For fixations on AOI 3, the same between-group effects were observed as for the first 3 s. Finally, the analysis of the AOI data for the first 5 s revealed no differences compared to the analysis of the first 4 s, and we argue that visuocognitive strategies stabilised within the first 4 s. Overall, the analyses suggest that experts use differentiated visual search strategies compared to the other two groups, which facilitates the information peak and processing, thus optimizing visuocognitive performance.

In terms of the AOIs, it is clear that the results for the initial phase of task processing for AOI 3 revealed the most between group differences. However, when considering complete trials, we conclude that AOI 2 offers the most promising area of interest for discriminating expertise-dependent differences in visuocognitive performance.

### Limitations

The study design has limitations. In order to examine them, the conduction of further experiments appears essential.

Firstly, we concede that despite the assumption that a fourth difficult trail could affect participants’ concentration, it should be considered that it can also lead to a reduction of data noises. From this point of view, further studies should make effort to experimentally evaluate the neurocognitive performance of chess players dependent on difficulty and the number of trials (e.g., fatigue effects).

Concerning the type of tasks, finding a sequence of moves leading to a mate is a straightforward task as the goal is clearly communicated (mate in “n” moves). This is a very specific and concrete task, and refers to the fact, that in chess, a mate is prepared by precise calculation. However, chess expertise is not only reflected by the ability to solve n-mate tasks. Hence, in order to win a chess match, a player must first develop a plan that will lead to material and/ or positional advantages. In our study design, the strategic planning of a chess game as a whole is eliminated and limited by the given situation to a few key moves only. From this point of view, we are unable to extend our findings to a global chess game progression.

Moreover, the chosen difficulty manipulation of the tasks might negatively affect the accurate detection of expertise-dependent visuocognitive strategies, as the more moves are required to solve the task, the more solution steps need to be considered and the more planning is required, followed by an intense visual search of the board. As a solution to this problem, two-move tasks with finely graded and increasing difficulty (e.g., piece constellation) might provide more accurate results.

Finally, the present research focused only on the visuocognitive approaches, ignoring the emotional states and traits of the participants, while limiting the multimodal understanding of chess play (Guntz et al., 2018). Therefore, the addition of more sensor technologies such as emotion detection (Guntz et al., 2018) and/ or mobile EEG registration (Katona, 2014) would provide more insight into the multimodal detection of chess playing procedures.

## Conclusion

The current state of chess research, concludes that expertise and visuo-cognitive performance is often limited on the one hand according to the fact that is very difficult to recruit chess experts and on the other due to the fact that the standardization of chess constellations should be based on “fiat” principles. As previously demonstrated, the present study employs a valid design which extents existing studies taking into account expertise, difficulty and at the same time a detailed spatial–temporal analysis of the participants’ visual searching strategies. In summary, our results help to identify crucial differences in the visuocognitive strategies of experts, intermediates and novices performing n-mate tasks in a close-to-natural chess environment. Superior visuocognitive performance, especially at the beginning of the response planning, and high processing efficiency require a high level of expertise. Effective visuocognitive strategies allow for a fast discrimination of task-specific relevant and irrelevant parts of the board, meaning that algorithms that incorporate such results could partly increase practitioners’ self-efficacy and confidence (Kővári and Katona, 2023). This can be achieved by manipulating task difficulty and increasing neurocognitive processing as a training effect. Moreover, visuocognitive performance in task processing is affected by the number of moves in which a mate can be executed, highlighting the importance of chess expertise. In this sense, the present findings provide a deeper insight into the visuocognitive advantages of experts and intermediates. They give impulses for future research concerning interrelations between visuocognition and planning of move sequences in given chess constellations. According to the results, we suggest that chess players should make an effort to extensively train their visuocognitive strategies (information peak and selection), for example by participating in *blitz chess*. This implies a decision-making under time pressure and optimising information filtering. Further research is needed in order to identify the critical time frames in which the most important information is processed and analysed in order to plan and execute strategic chess moves. Moreover, it will be interesting if future investigations could focus on the relationship between age effects and visuocognitive chess.

## Data Availability

The raw data supporting the conclusions of this article will be made available by the authors, without undue reservation.

## Footnotes

^1^An exhaustive review about how cognitive science has been augmented by chess research is beyond the scope of this article. For the great impact of chess research on cognitive science, please see Charness (1992).

^2^DWZ = *Deutsche Wertungszahl* – *German rating number* which corresponds to the international ELO rating system.
