# Supplementary material for: Expertise-dependent visuocognitive performance of chess players in mating tasks: evidence from eye movements during task processing
Source: Front Psychol. 2024 Oct 24;15:1294424. doi: 10.3389/fpsyg.2024.1294424 (PMC11540784; doi:10.3389/fpsyg.2024.1294424)

All chess tasks with corresponding Areas Of Intererst (*AOI*s)

AOI 1 (red) includes the attacked king, the attacker(s) and destination square(s) of the attacking move(s).
AOI 2 includes AOI 1 plus piece replacement squares (yellow) of the attacker(s).
AOI 3 includes AOI 1 and AOI 2 plus nearest neighbour squares (green) of all squares which constitute AOI 1.


**Task 1**


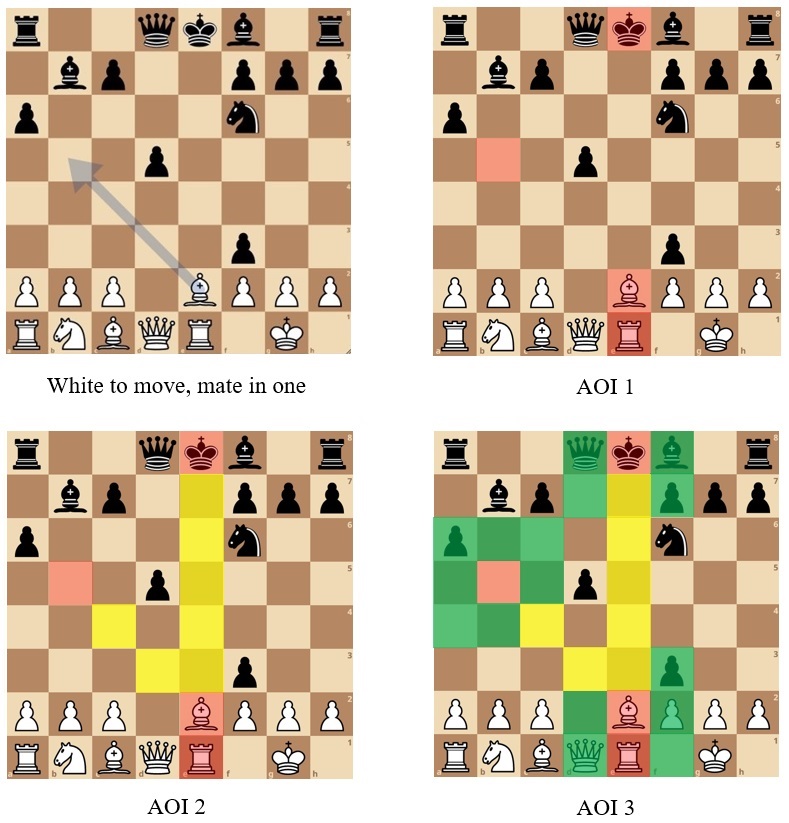


**Task 2**


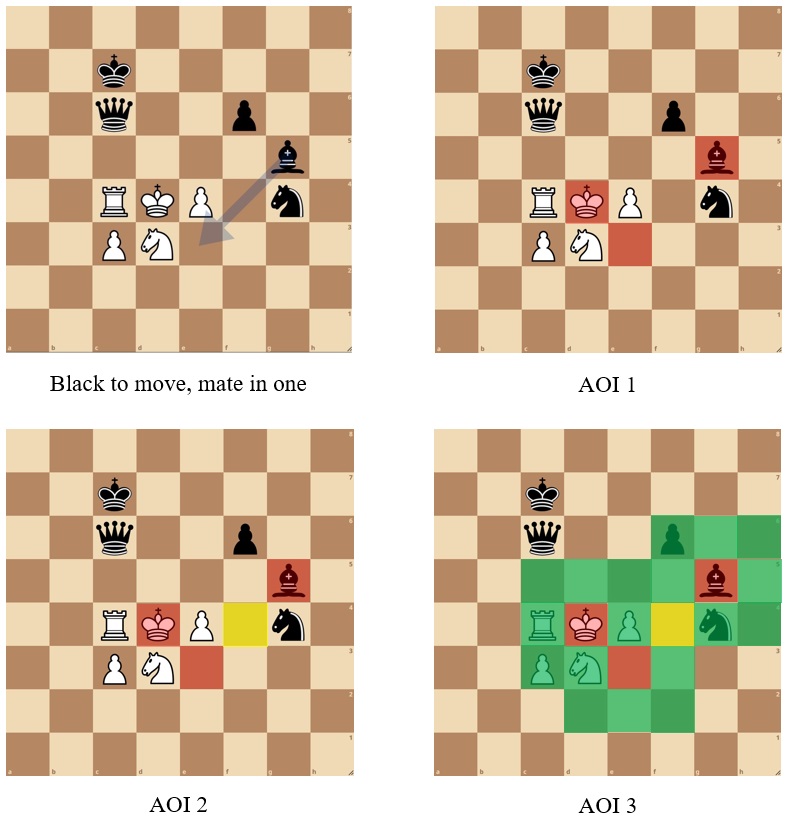


**Task 3**


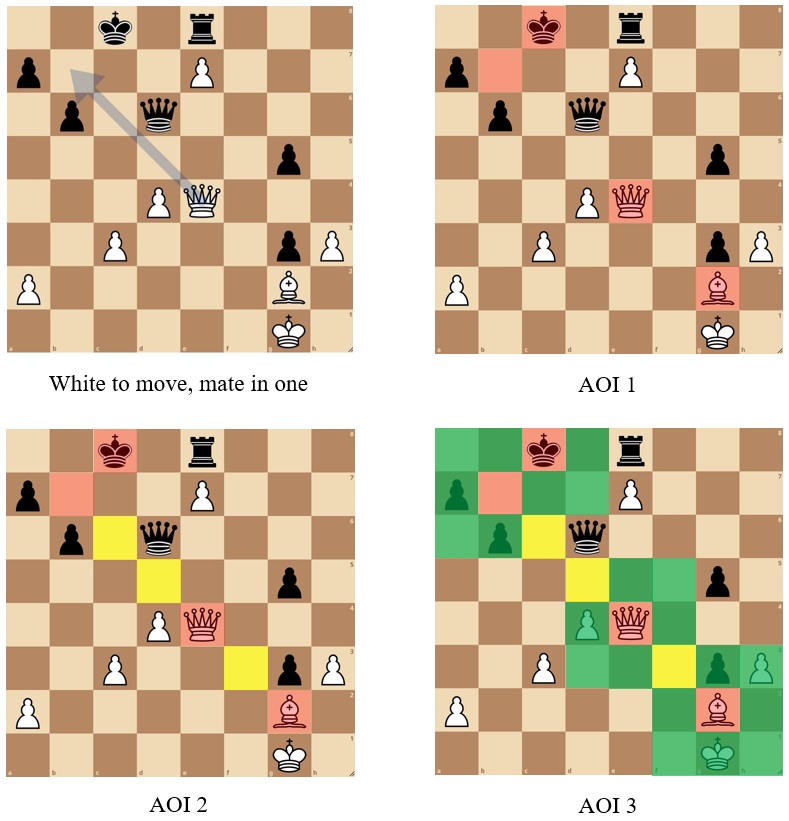

**Task 4**


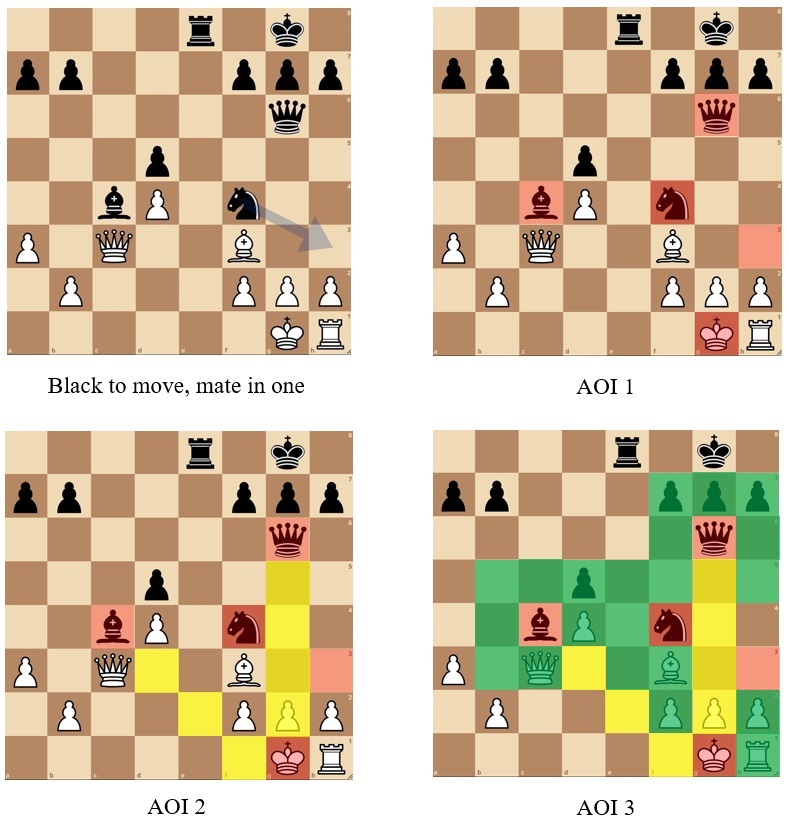


**Task 5**


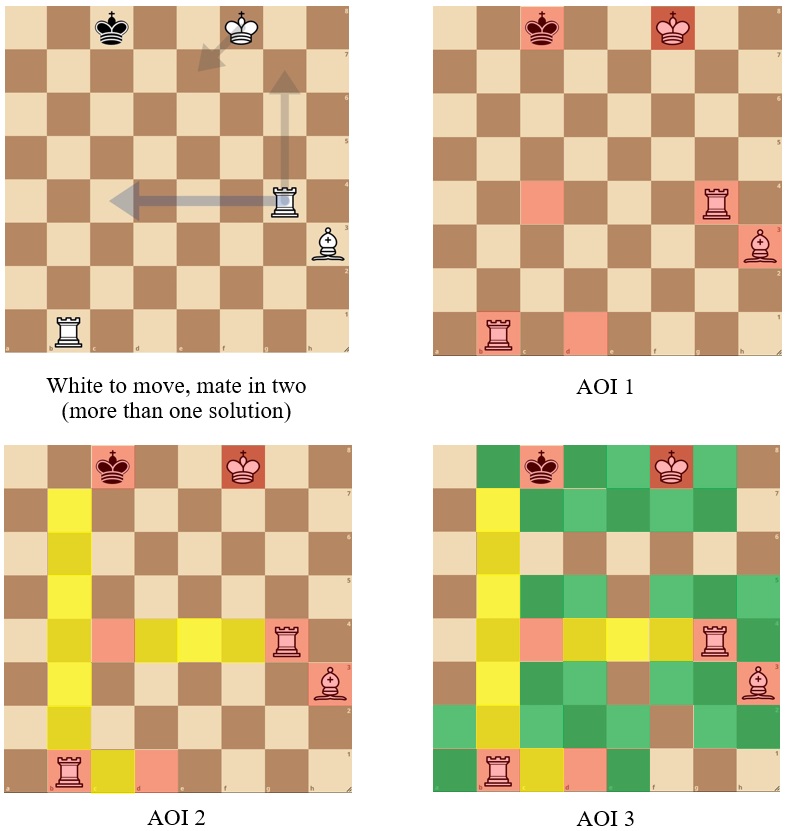

**Task 6**


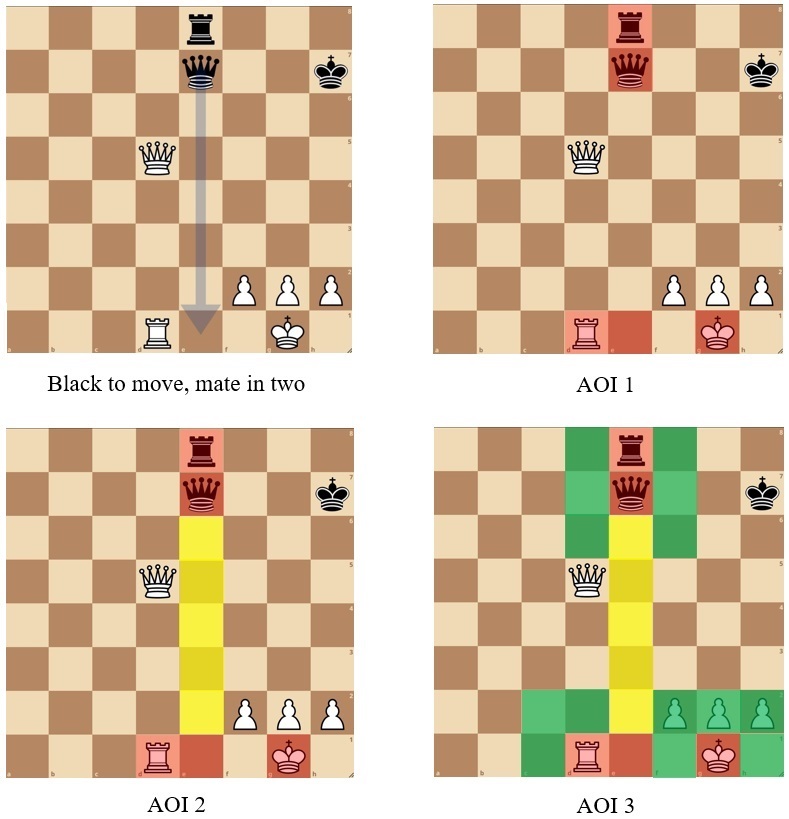


**Task 7**


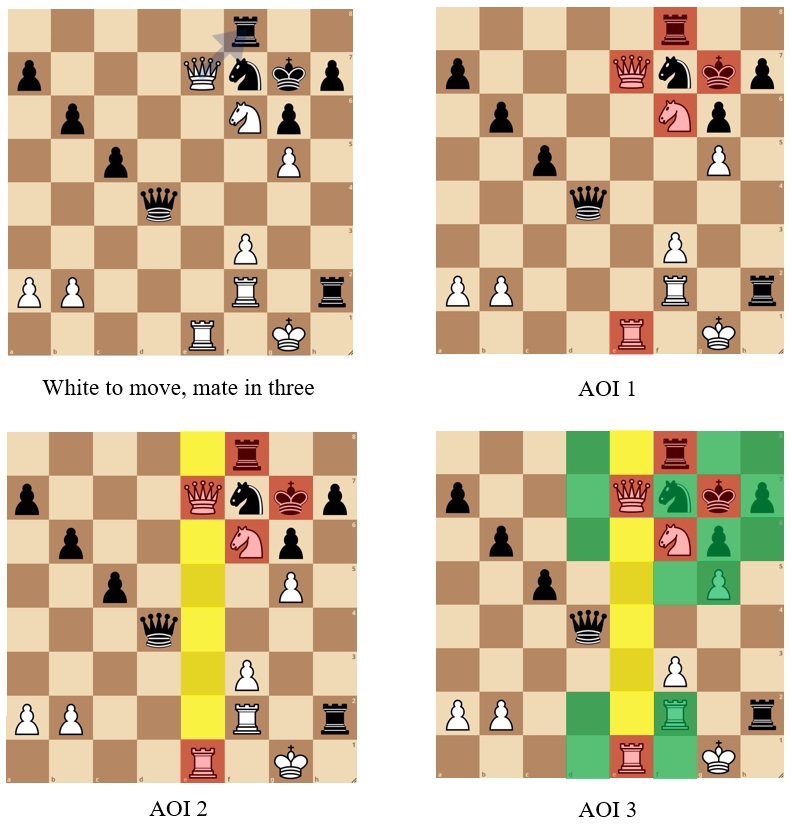

**Task 8**


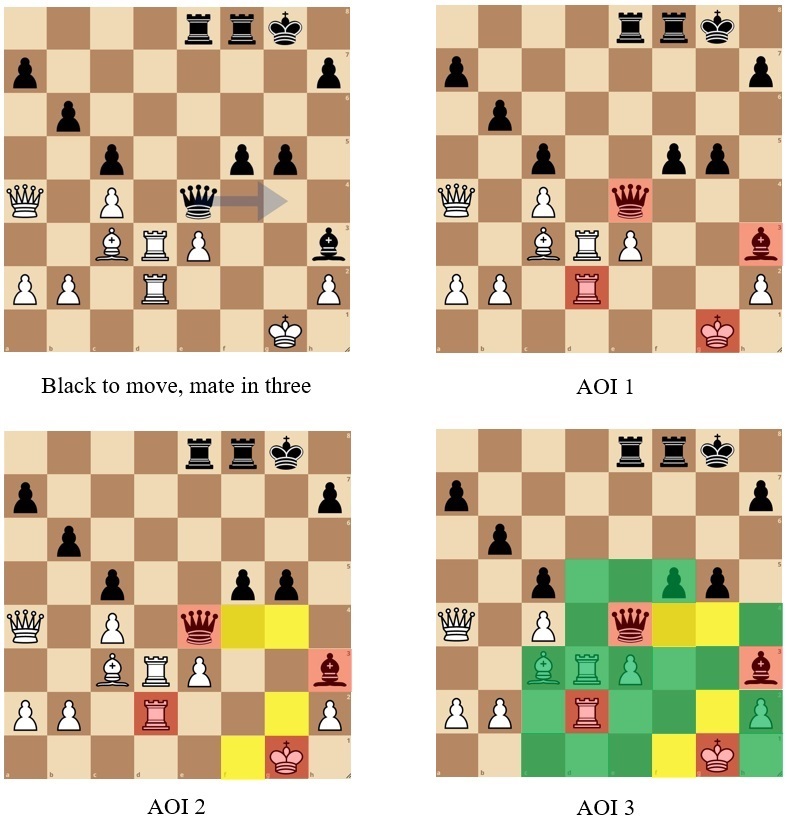


**Task 9**


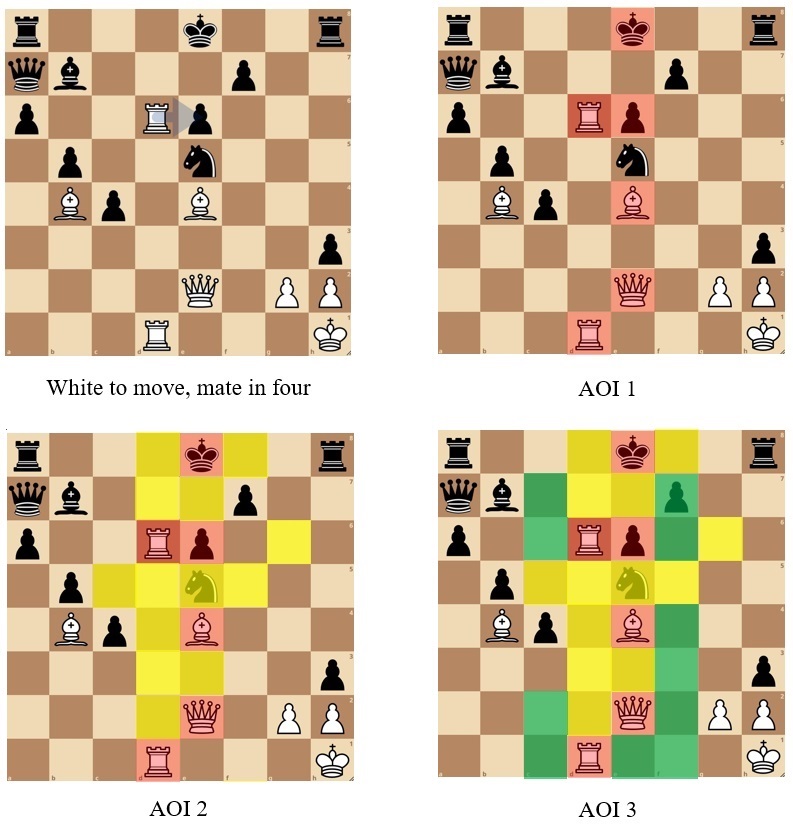

**Task 10**


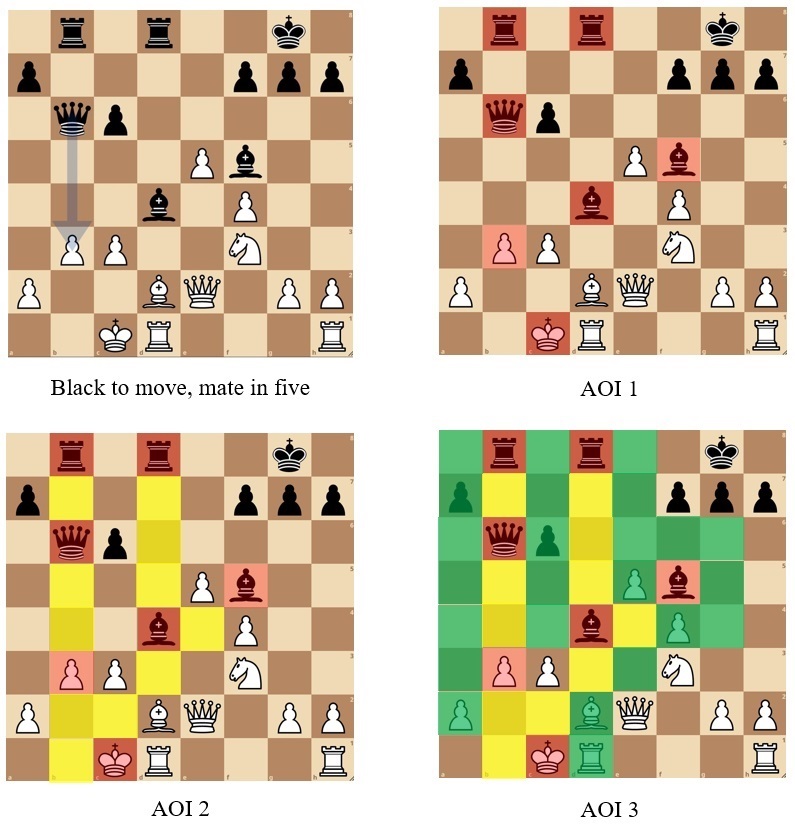


**Task 11**


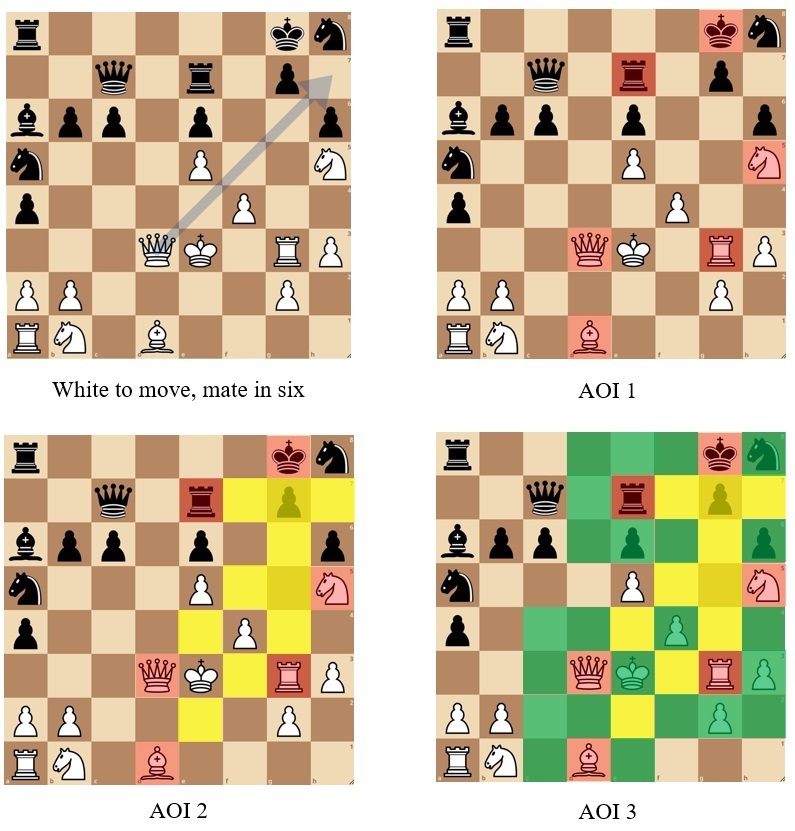

Supplement: Supplementary file 1 [file Table_1.DOCX]
